# Supplementary material for: Organocatalytic Enantioselective Michael Reaction of Aminomaleimides with Nitroolefins Catalyzed by Takemoto’s Catalyst
Source: Molecules. 2022 Nov 12;27(22):7787. doi: 10.3390/molecules27227787 (PMC9696348; doi:10.3390/molecules27227787)
Supplement: Supplementary file 1 [file molecules-27-07787-s001.zip › molecules-2023061-supplementary.pdf]

Supporting Information

Oragnocatalytic Enantioselective Michael Reaction of Aminomaleimides with Nitroolefins Catalyzed by Takemoto's catalyst

Hongwen Mu[1,2], Yan Jin[2], Rongrong Zhao[1], Liming Wang[1*], Ying Jin[1,2*]

[1]*Department of Pharmacy, Jilin Medical University, Jilin, Jilin 132013, China*

[2]*Yanbian University, Yanji, Jilin 133000, China*

^{1}H NMR and ^{13}C NMR spectra… ……………………...…………….........…S2–S16

HPLC trace.....……………...…………………………...………........…..... S17–S36

^1H NMR and ^{13}C NMR spectra

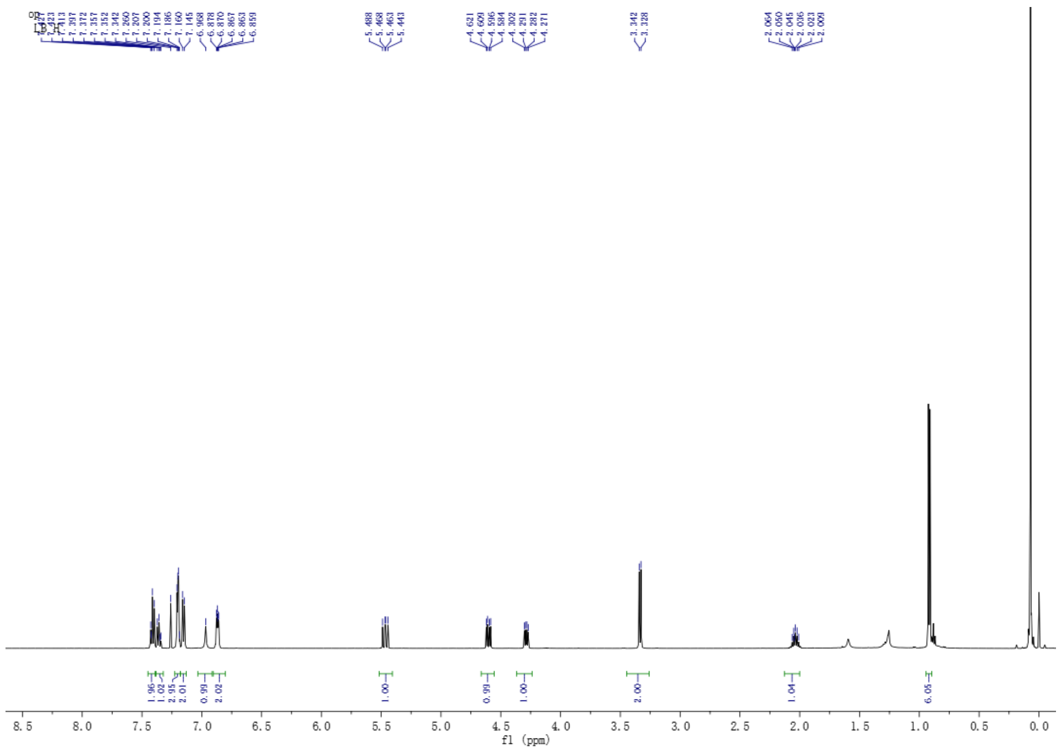

Figure S1. ^1H NMR spectrum of **4a**

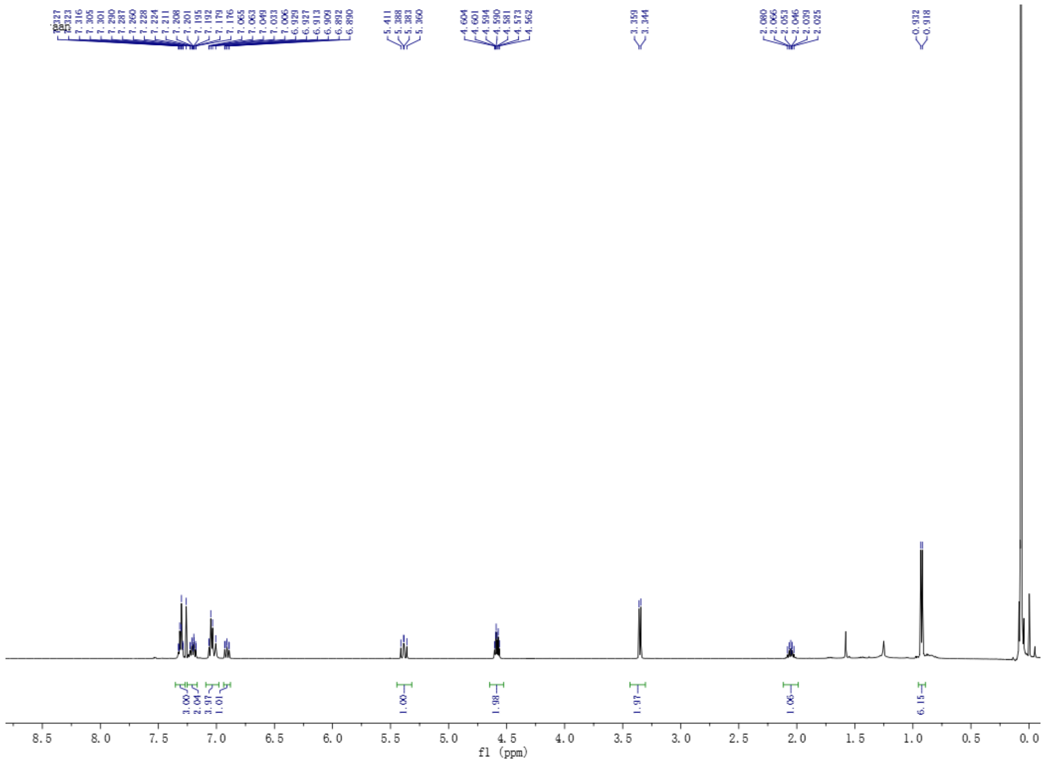

Figure S2. ^{1}H NMR spectrum of **4b**

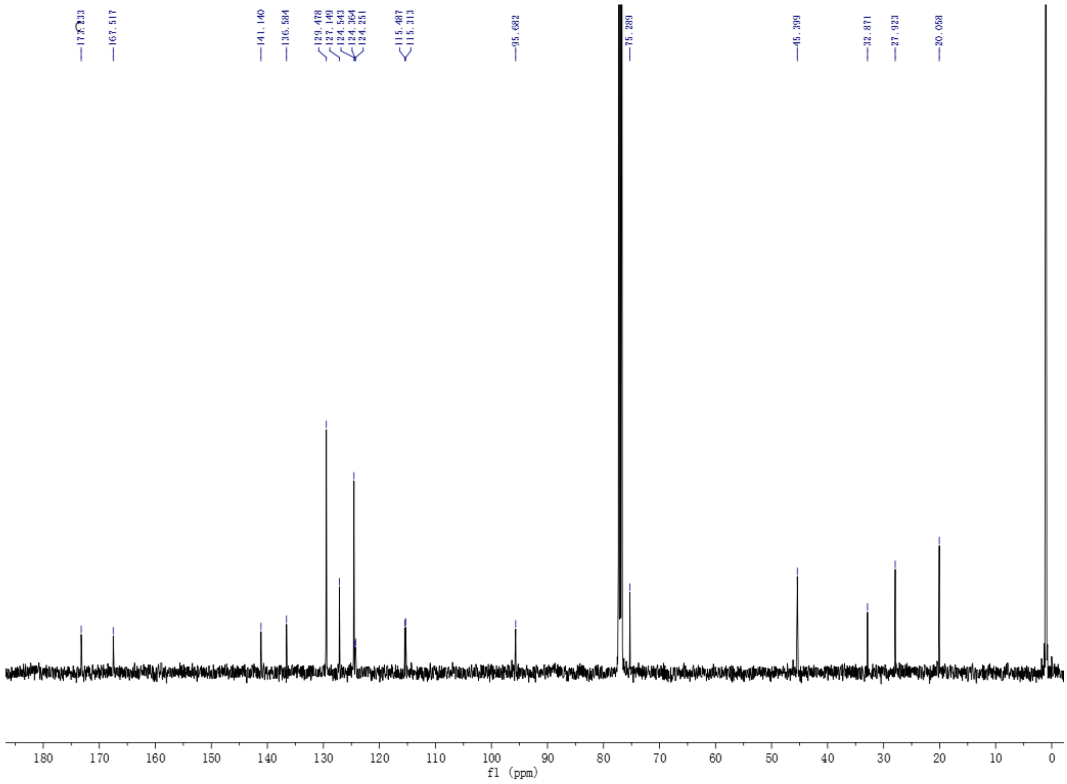

Figure S3. ^{13}C NMR spectrum of **4b**

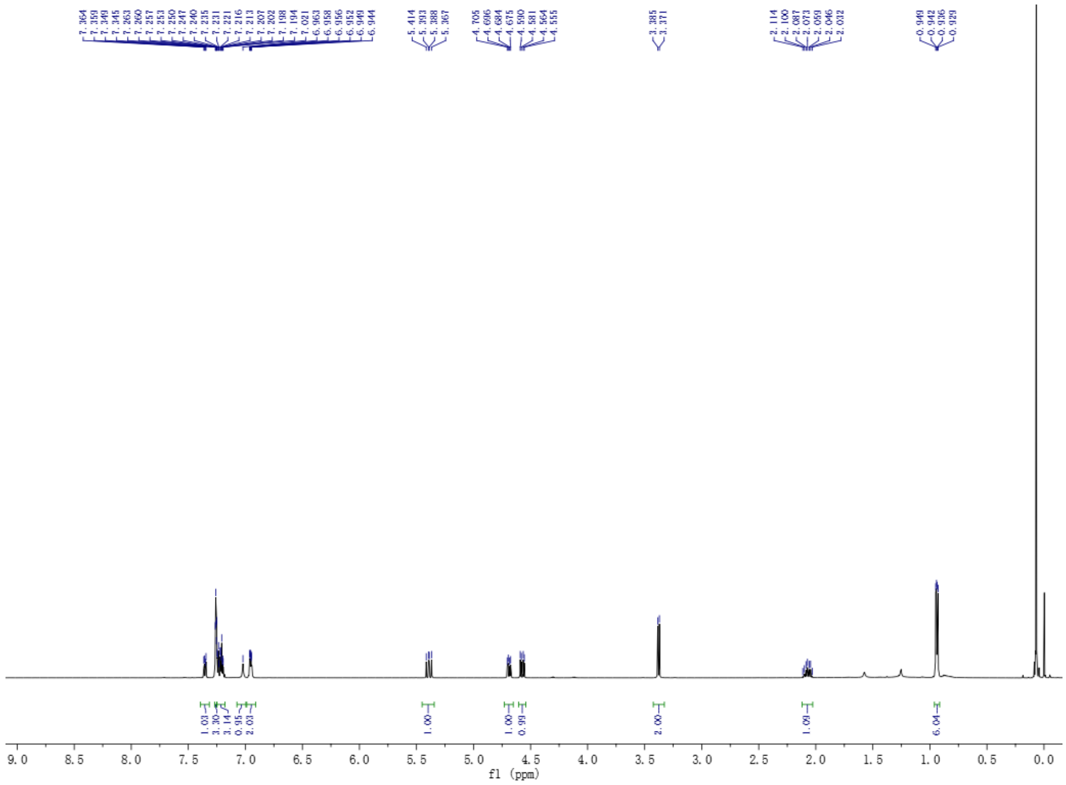

Figure S4. ^{1}H NMR spectrum of **4c**

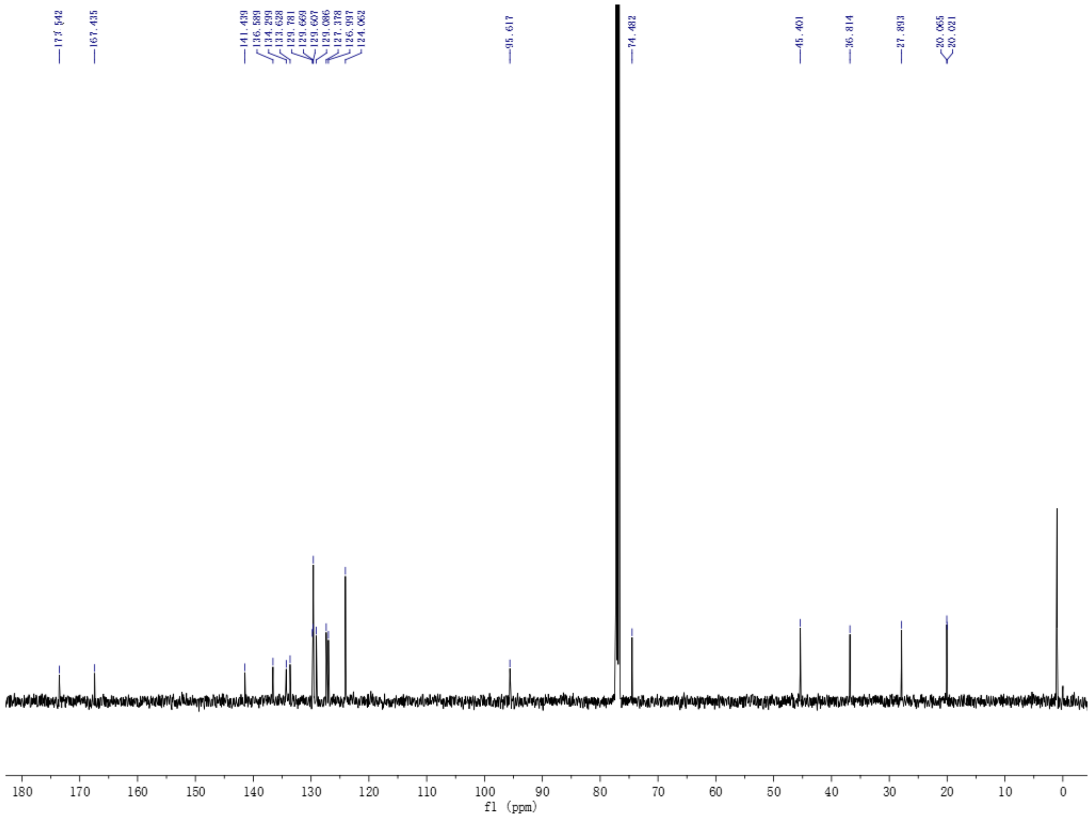

Figure S5. ^{13}C NMR spectrum of **4c**

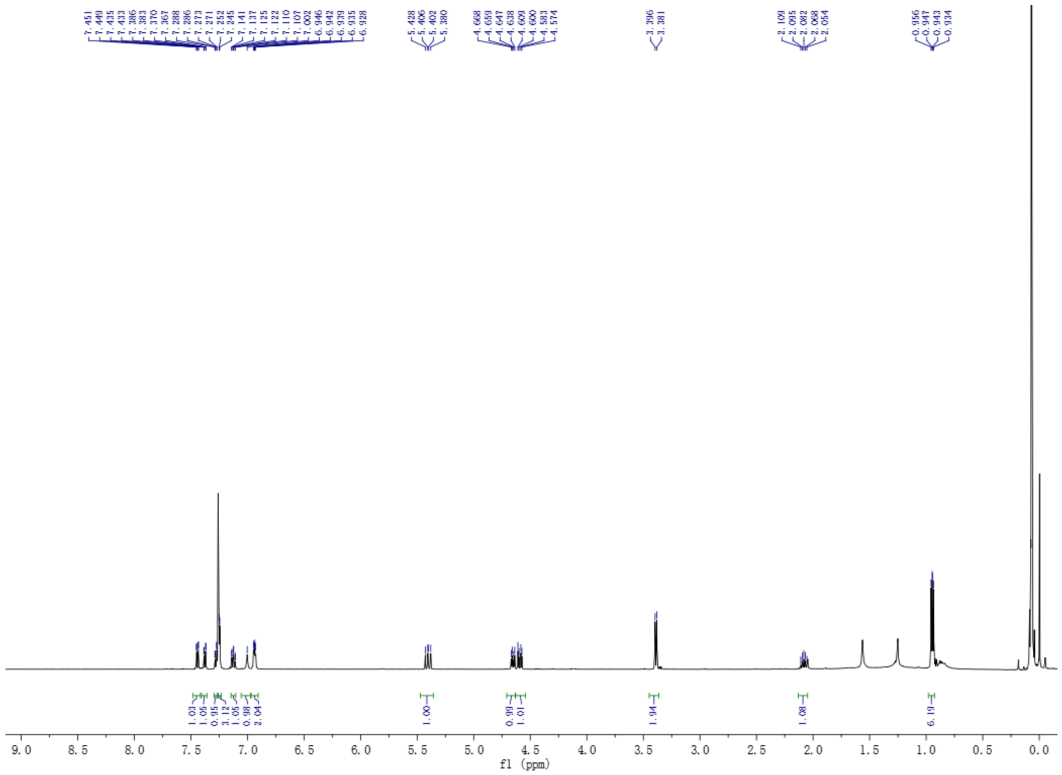

Figure S6. ^{1}H NMR spectrum of **4d**

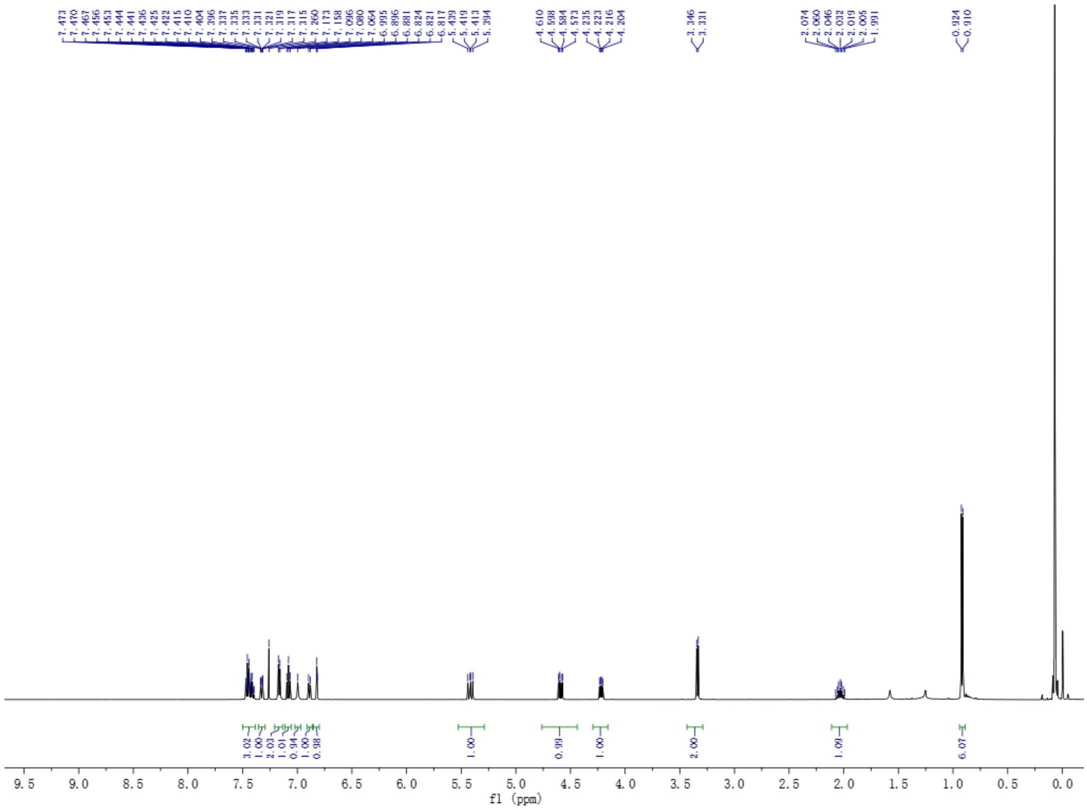

11

Figure S7. ^{1}H NMR spectrum of **4e**

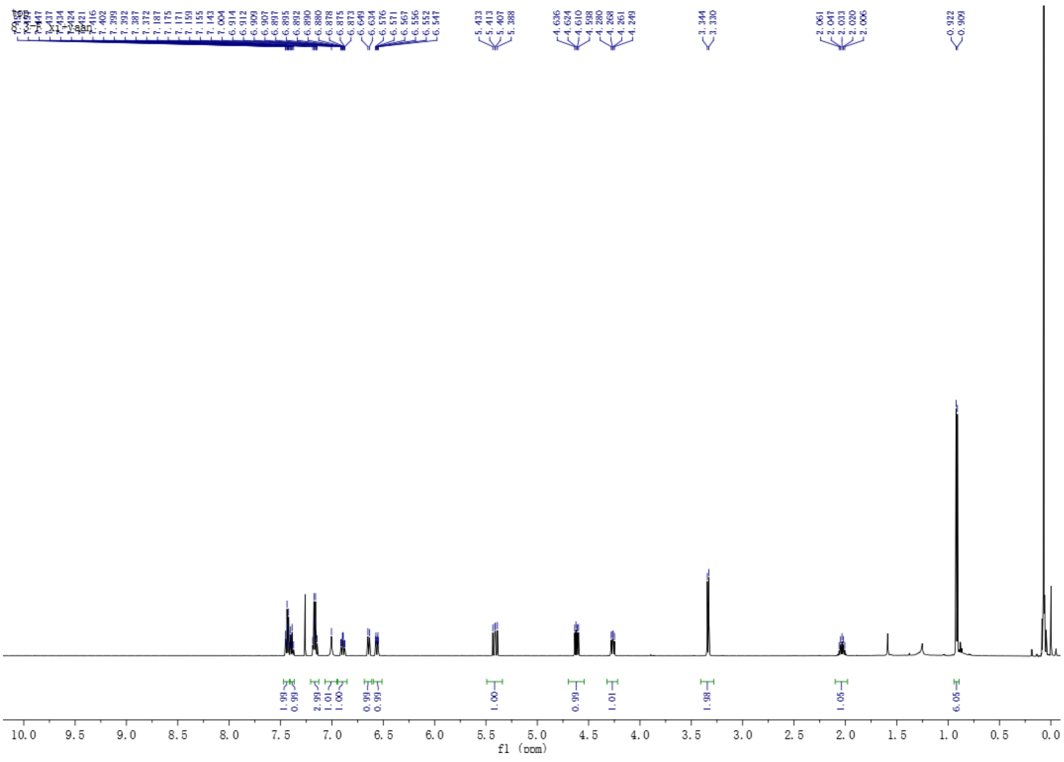

Figure S8. ^{1}H NMR spectrum of **4f**

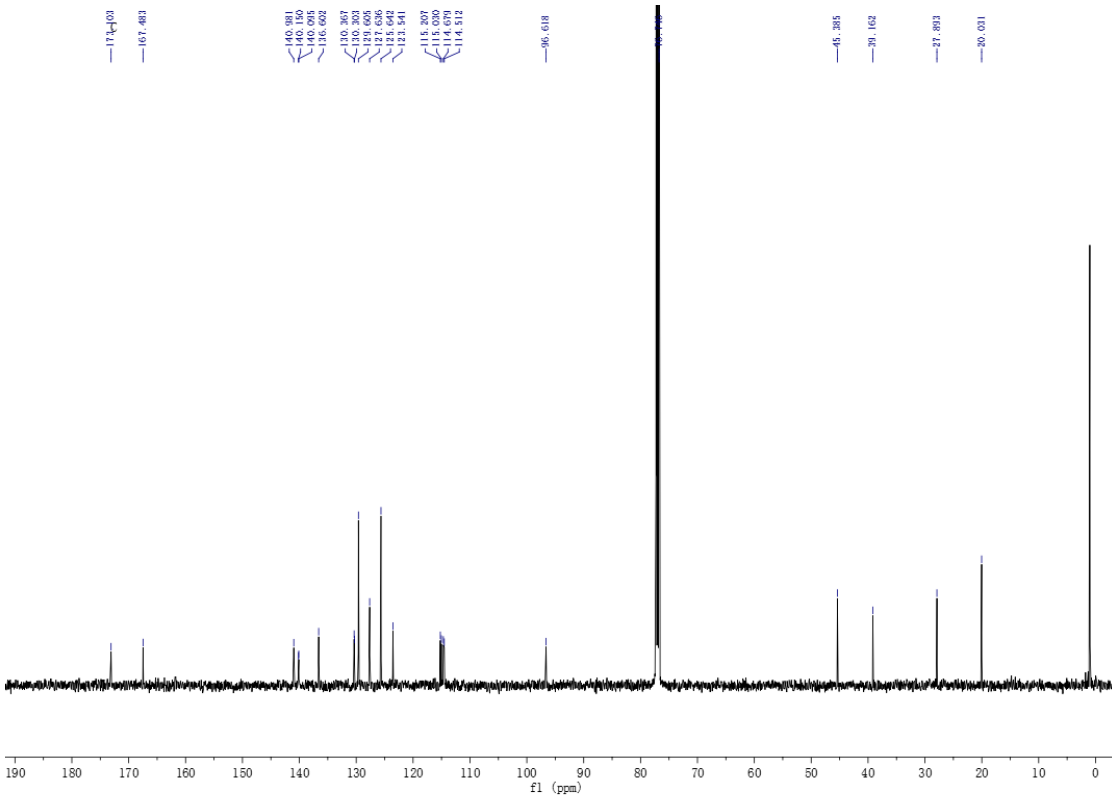

Figure S9. ^{13}C NMR spectrum of **4f**

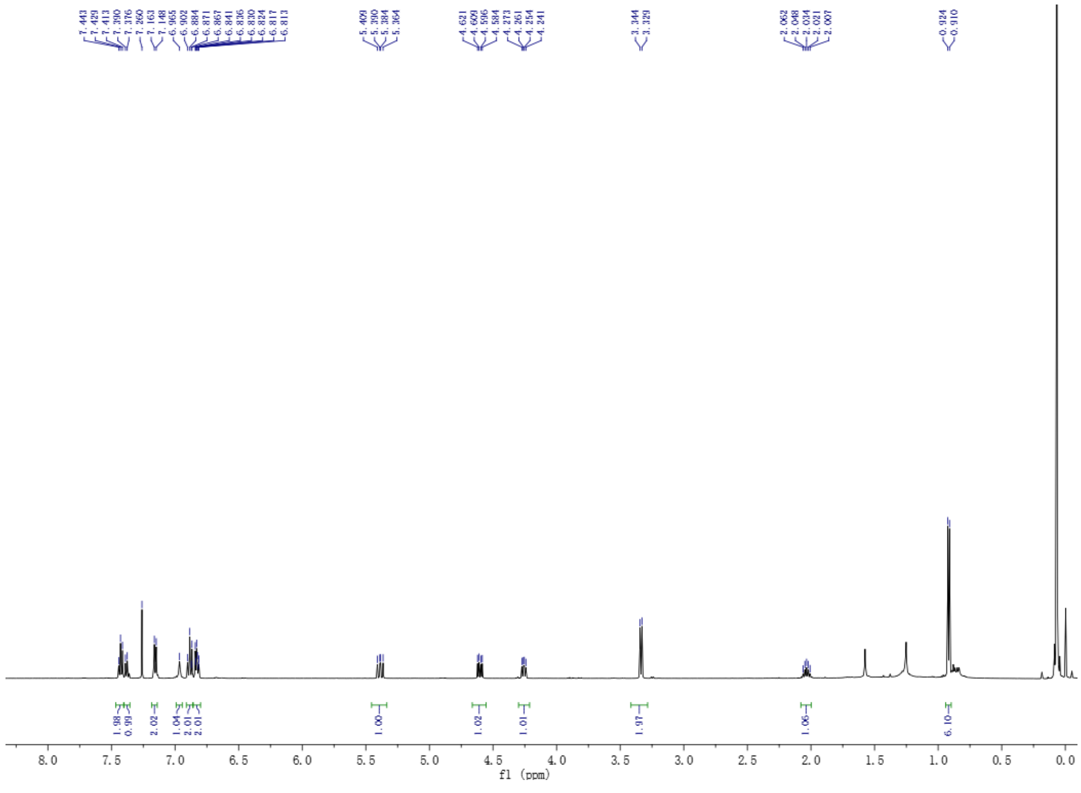

Figure S10 ^{1}H NMR spectrum of **4g**

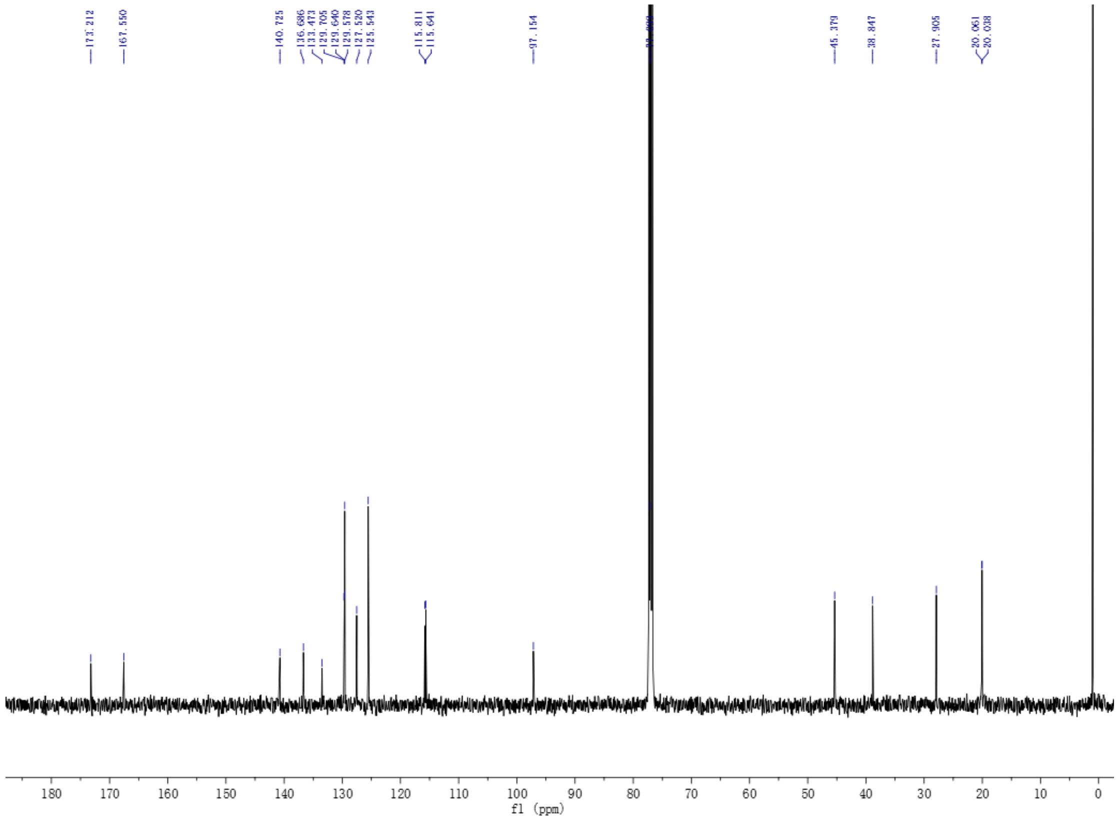

Figure S11. ^{13}C NMR spectrum of **4g**

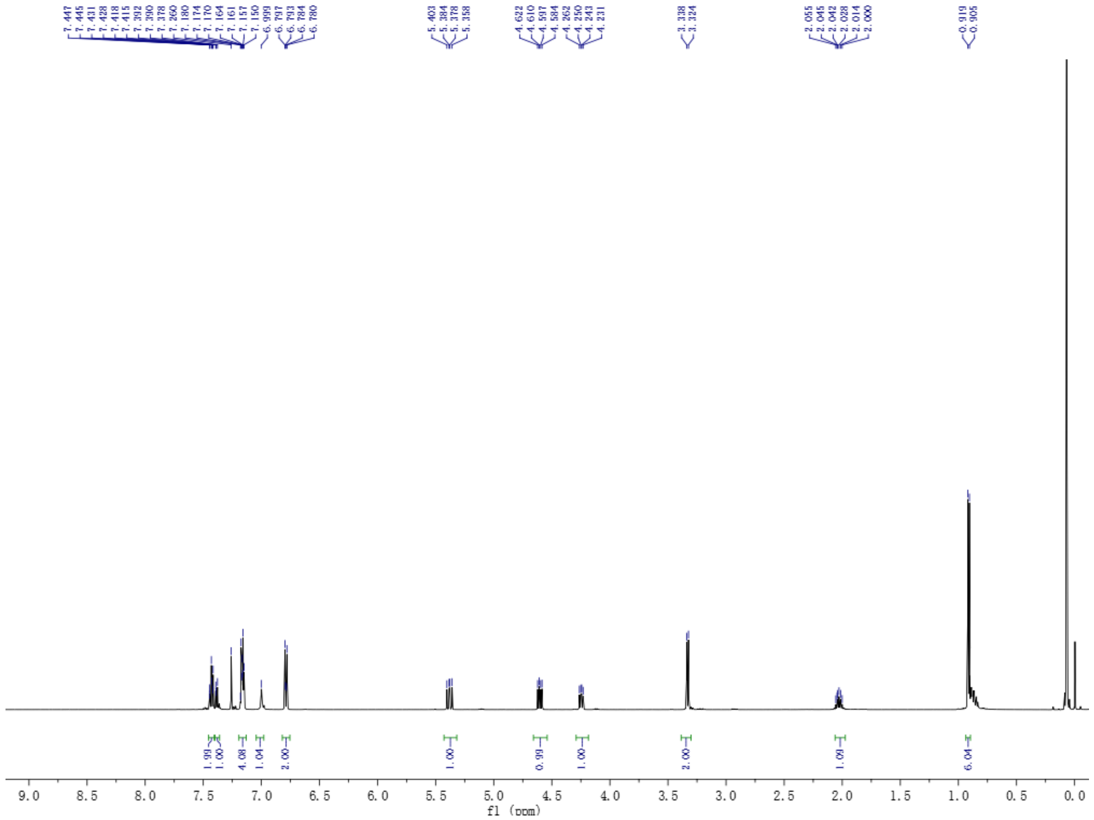

Figure S12. ^{1}H NMR spectrum of **4h**

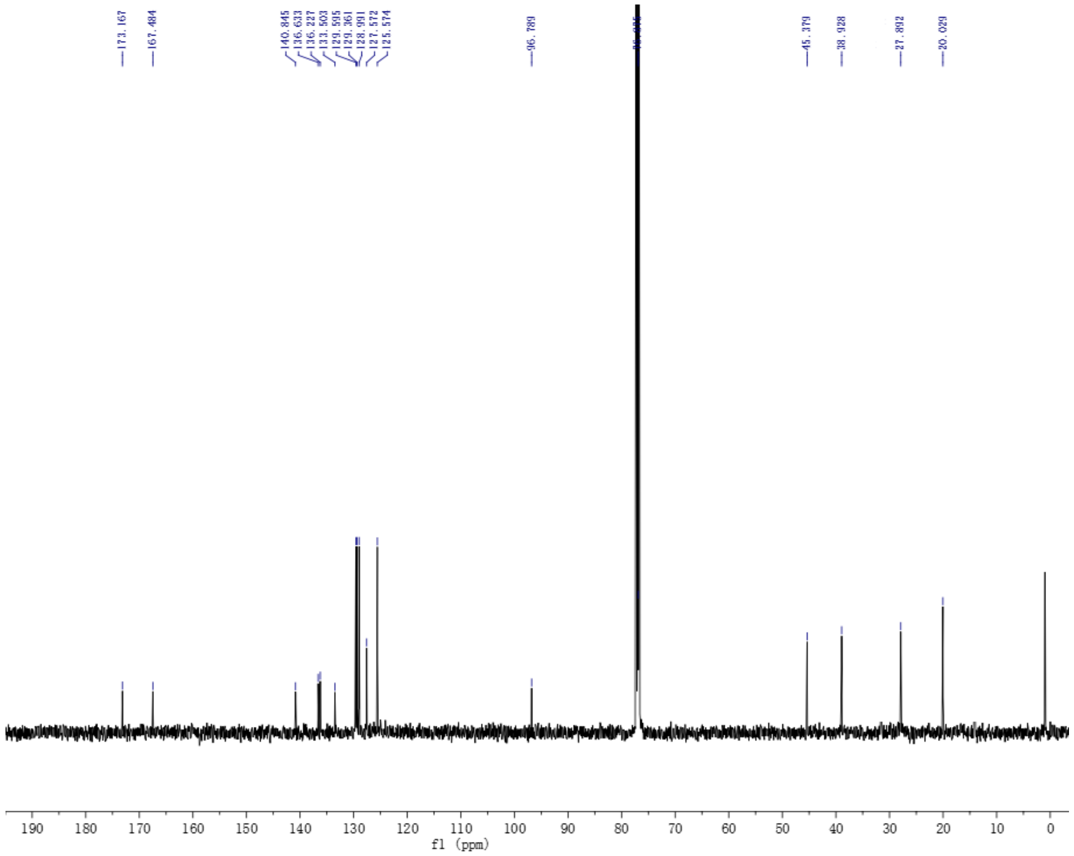

Figure S13. ^{13}C NMR spectrum of **4h**

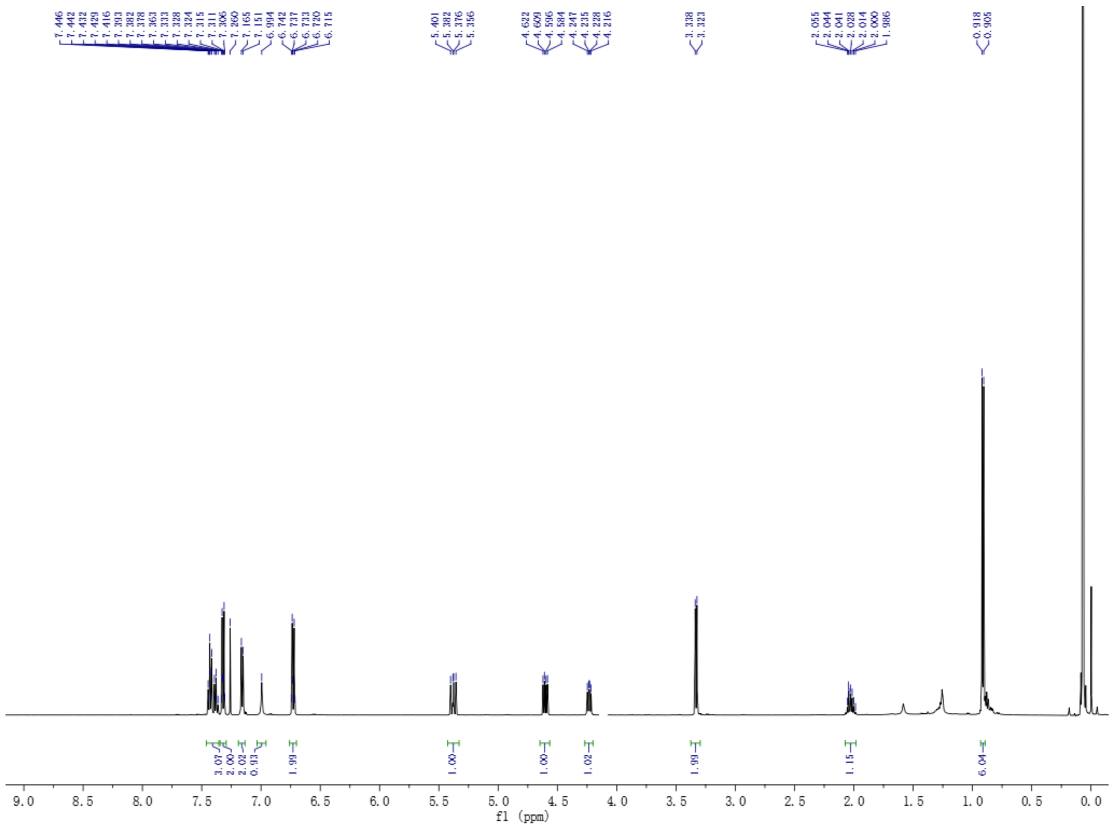

Figure S14. ^{1}H NMR spectrum of **4i**

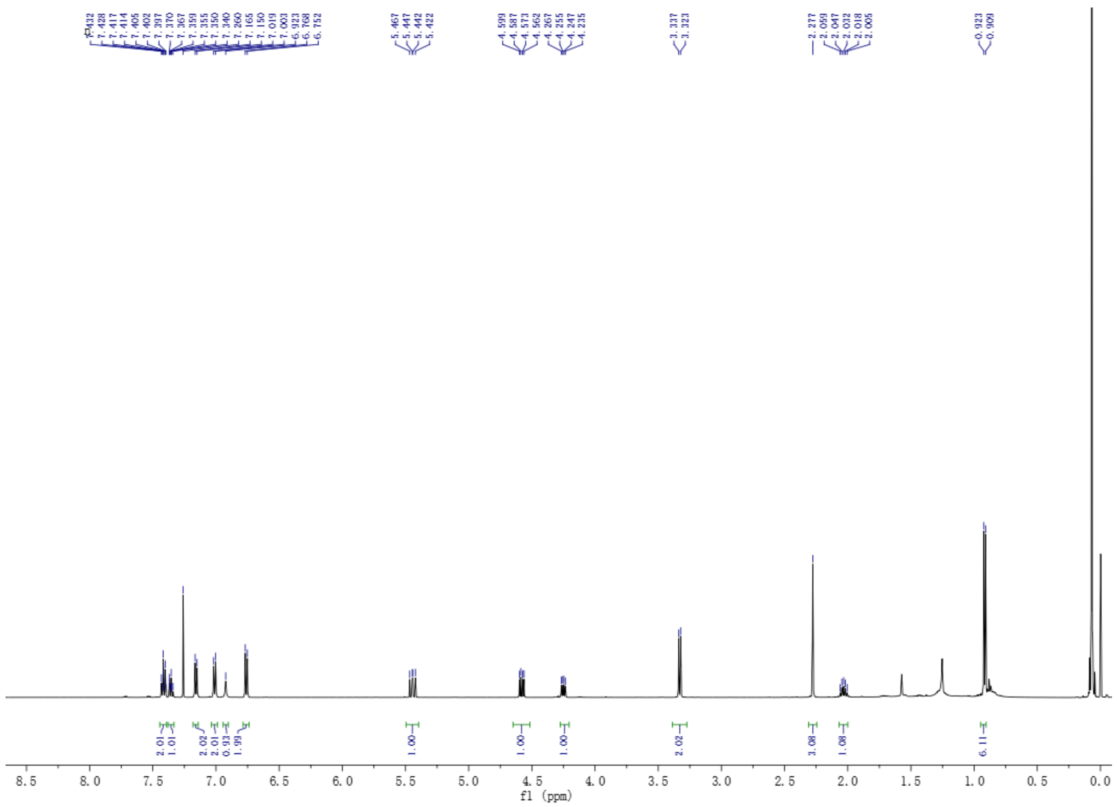

Figure S15. ^{1}H NMR spectrum of **4j**

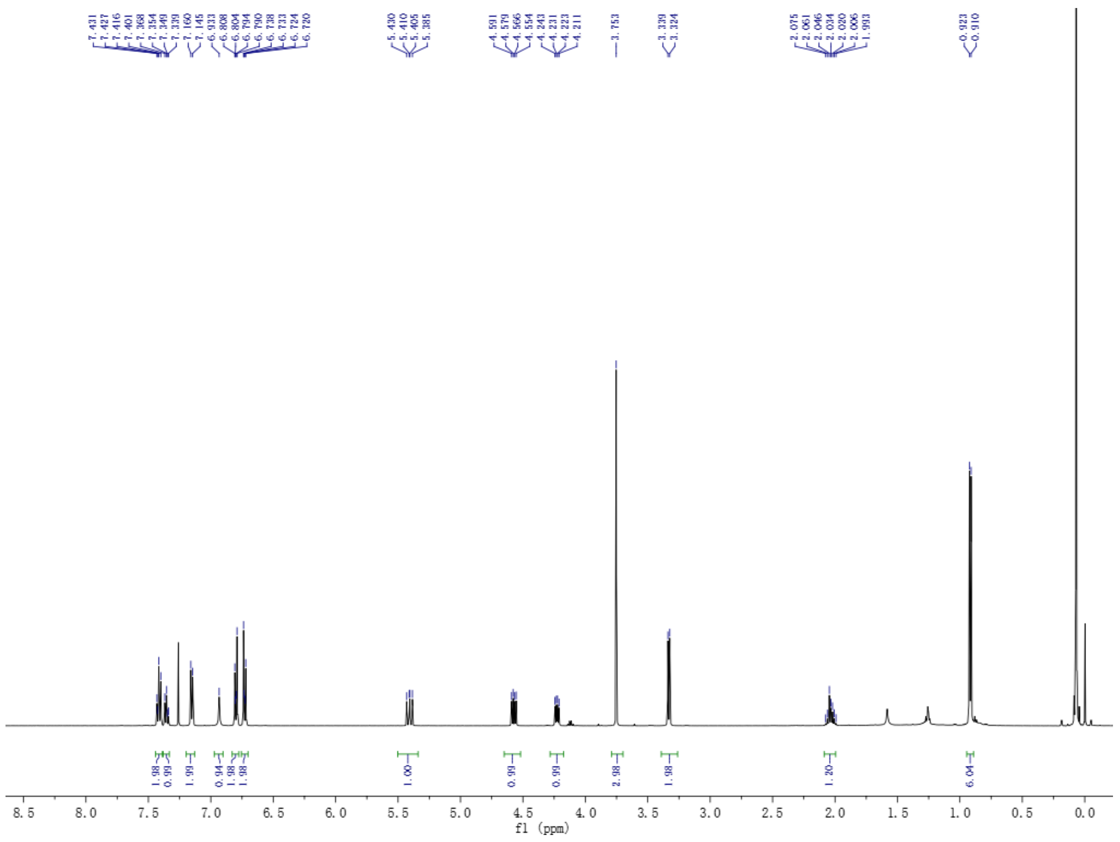

Figure S16. ^{1}H NMR spectrum of **4k**

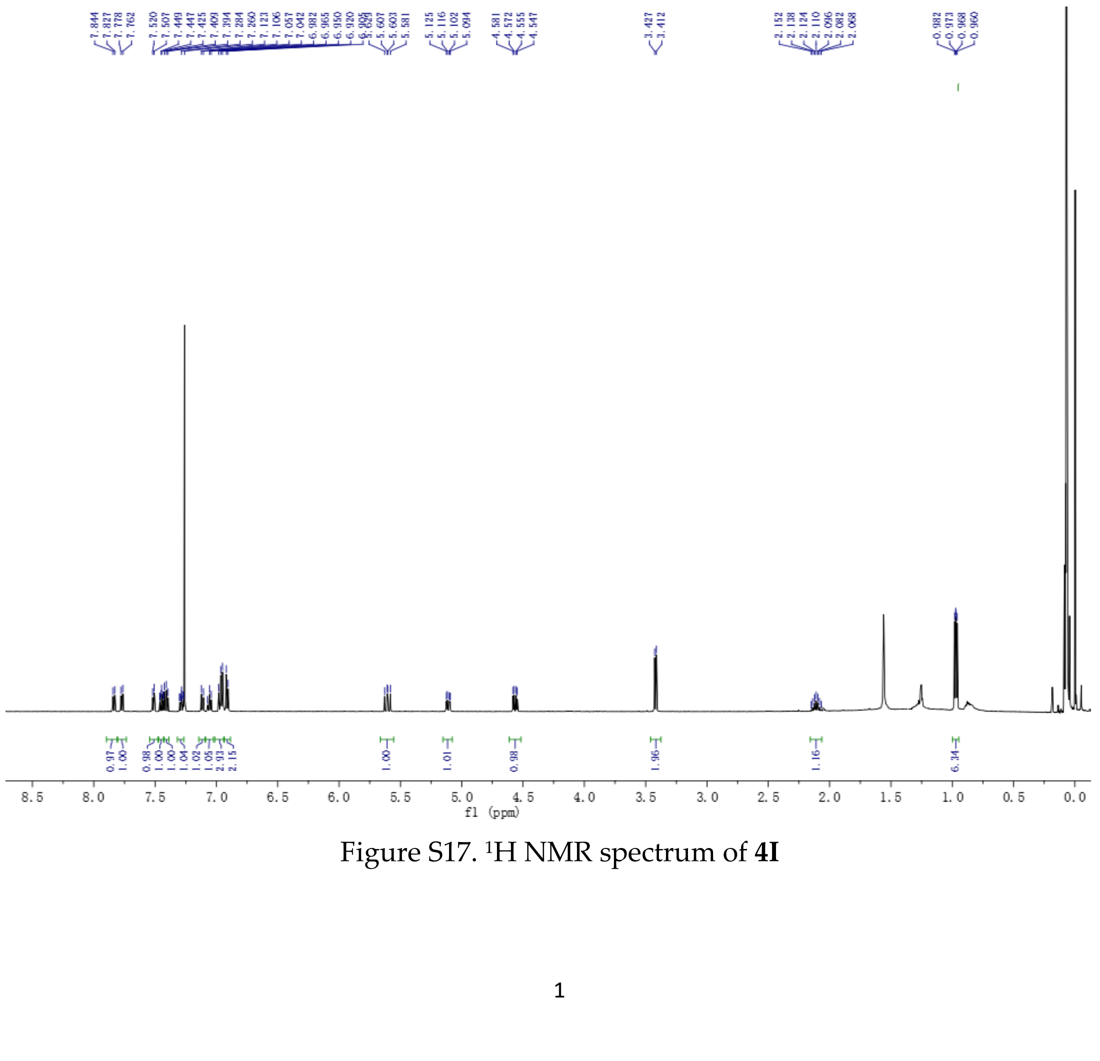

Figure S17. ^{1}H NMR spectrum of **4l**

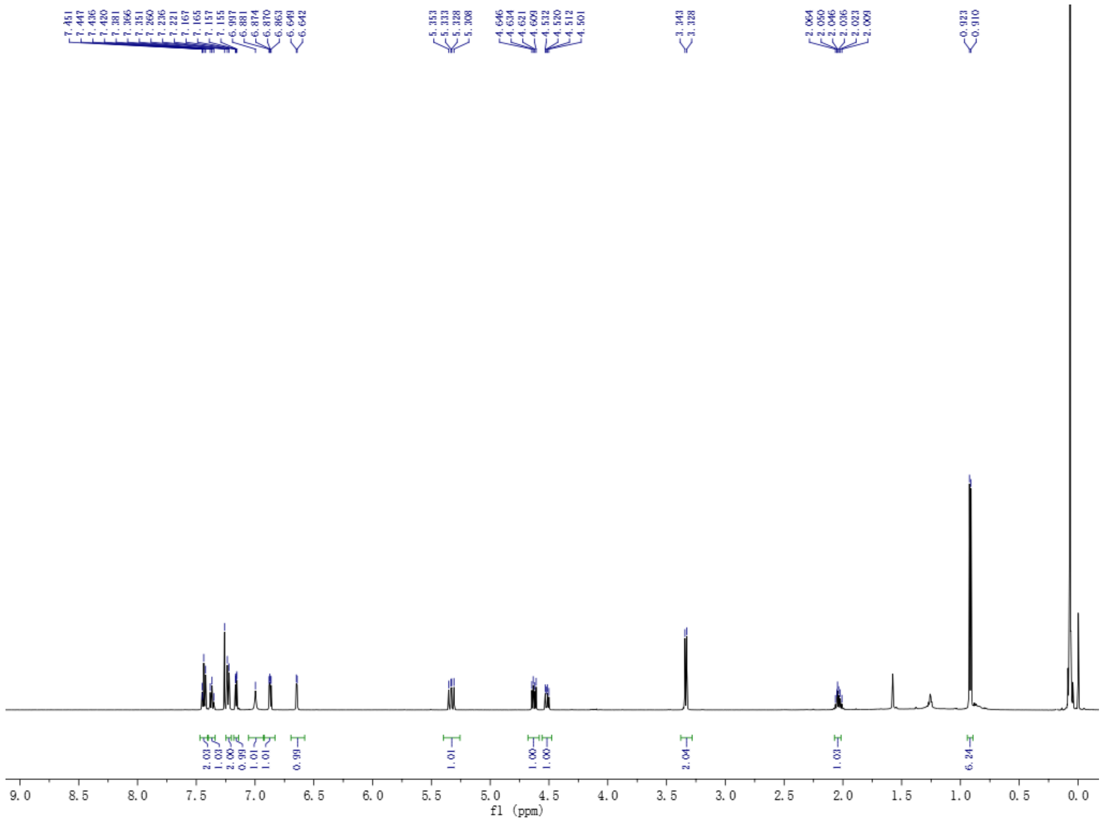

Figure S18. ^{1}H NMR spectrum of **4m**

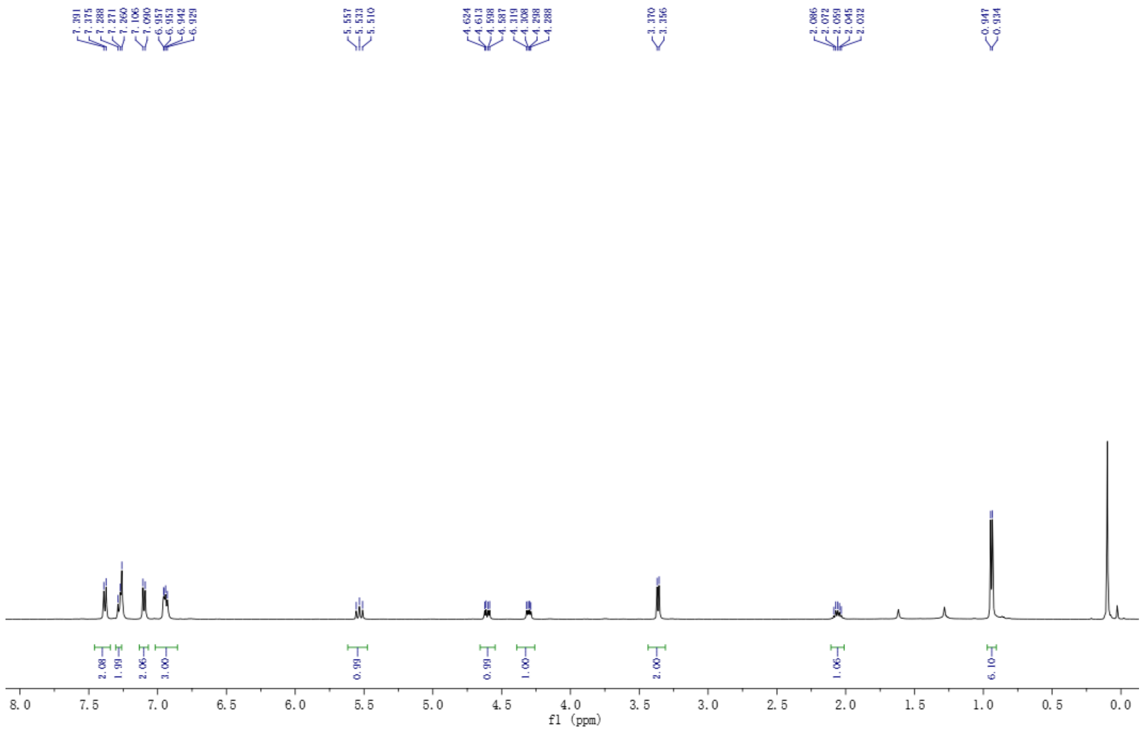

Figure S19. ^{1}H NMR spectrum of **4n**

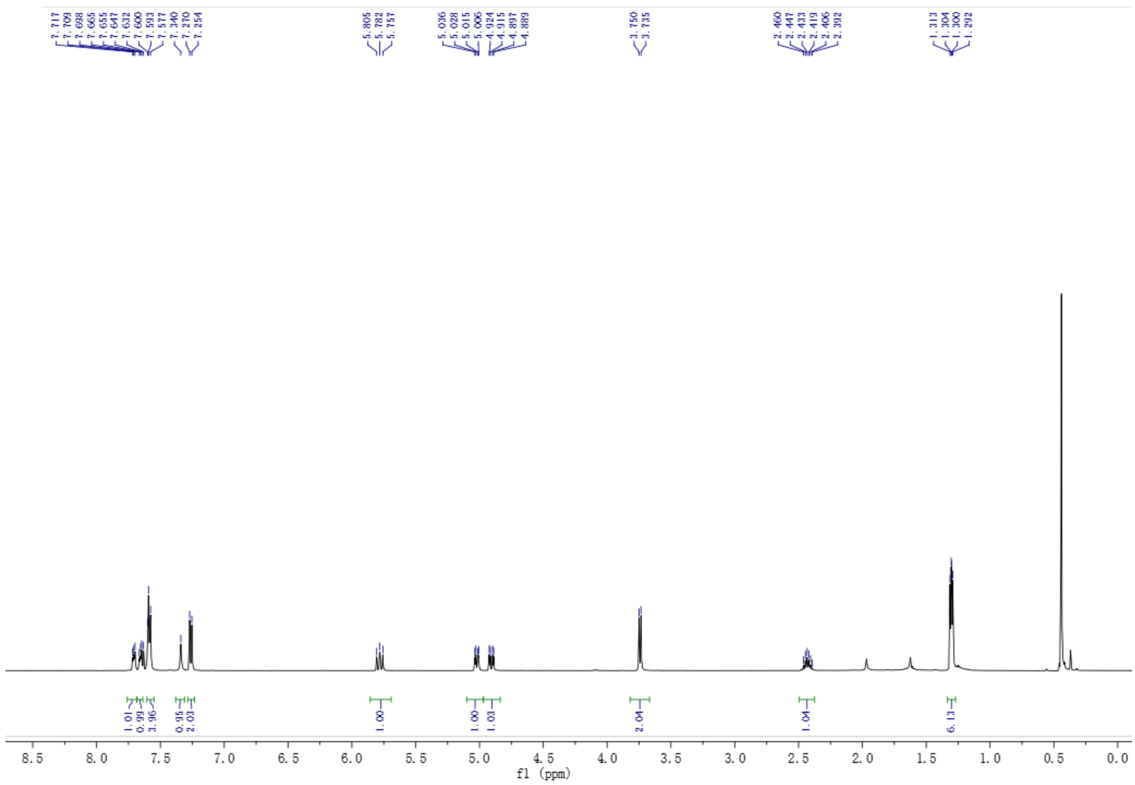

Figure S20. ^{1}H NMR spectrum of **4o**

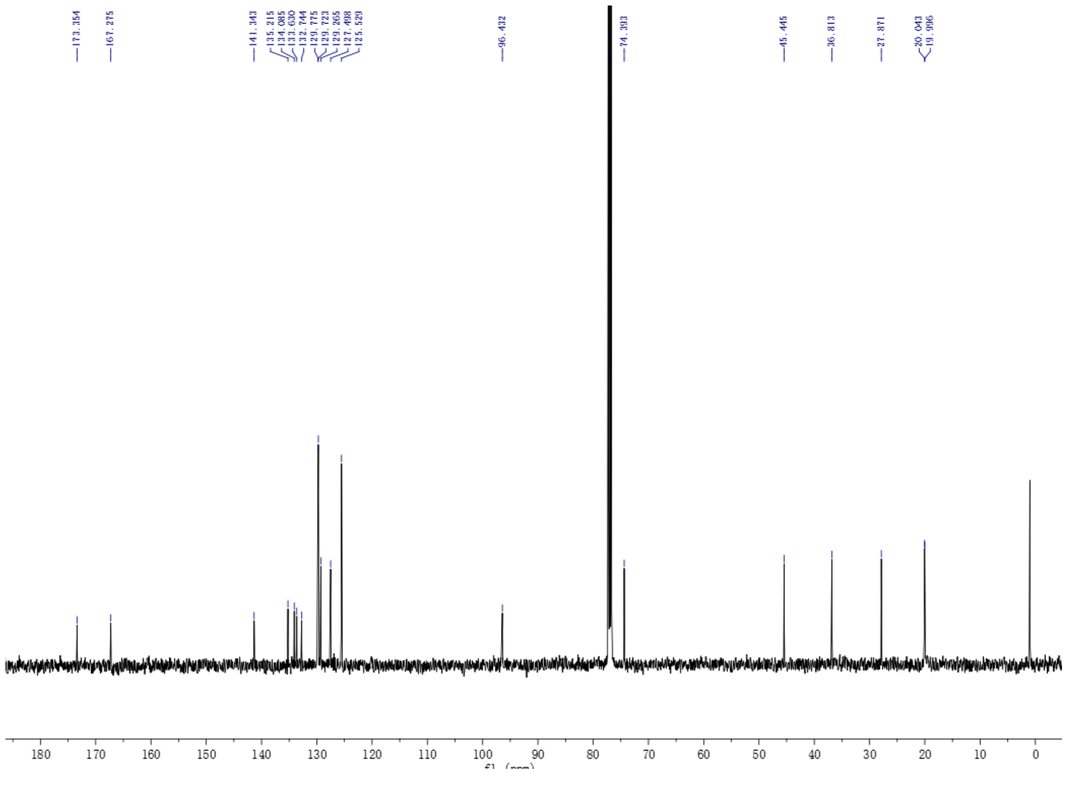

Figure S21. ^{13}C NMR spectrum of **4o**

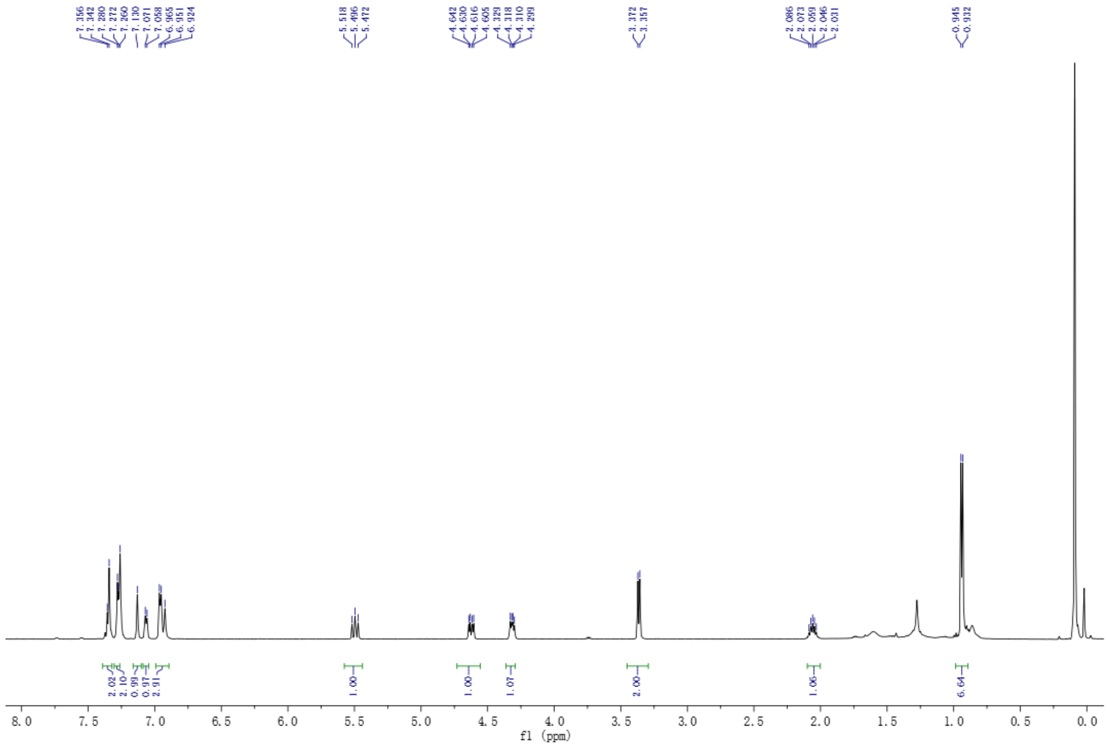

Figure S22. ^{1}H NMR spectrum of **4p**

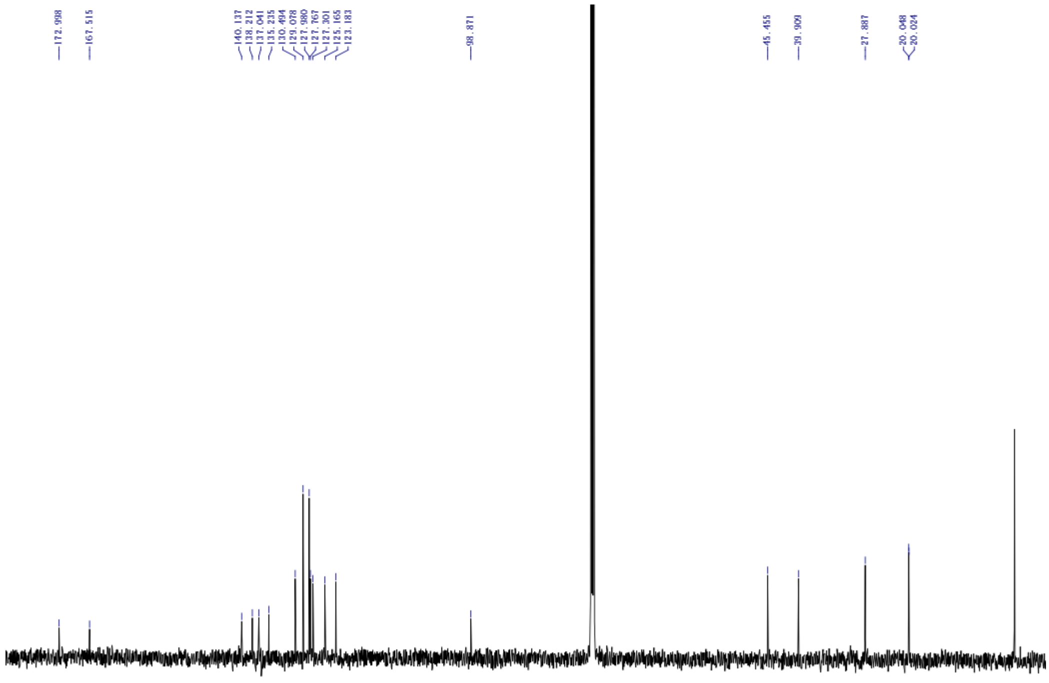

Figure S23. ^{13}C NMR spectrum of **4p**

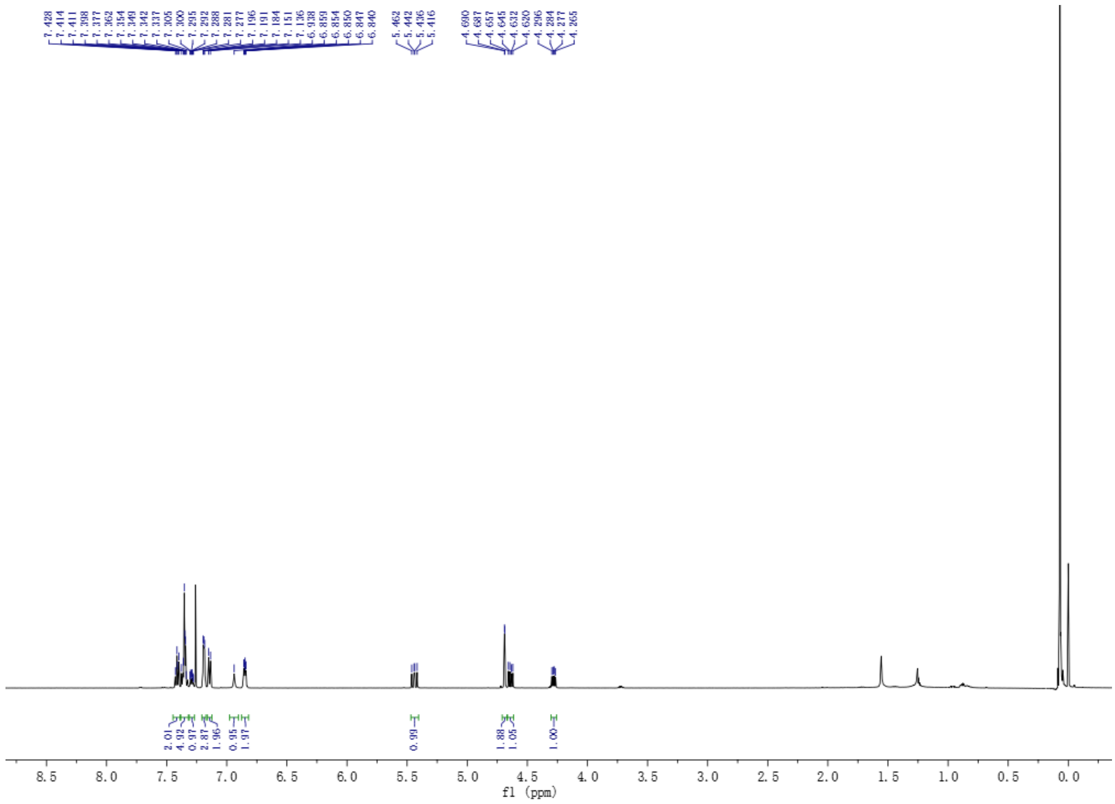

Figure S24. ^{1}H NMR spectrum of **4q**

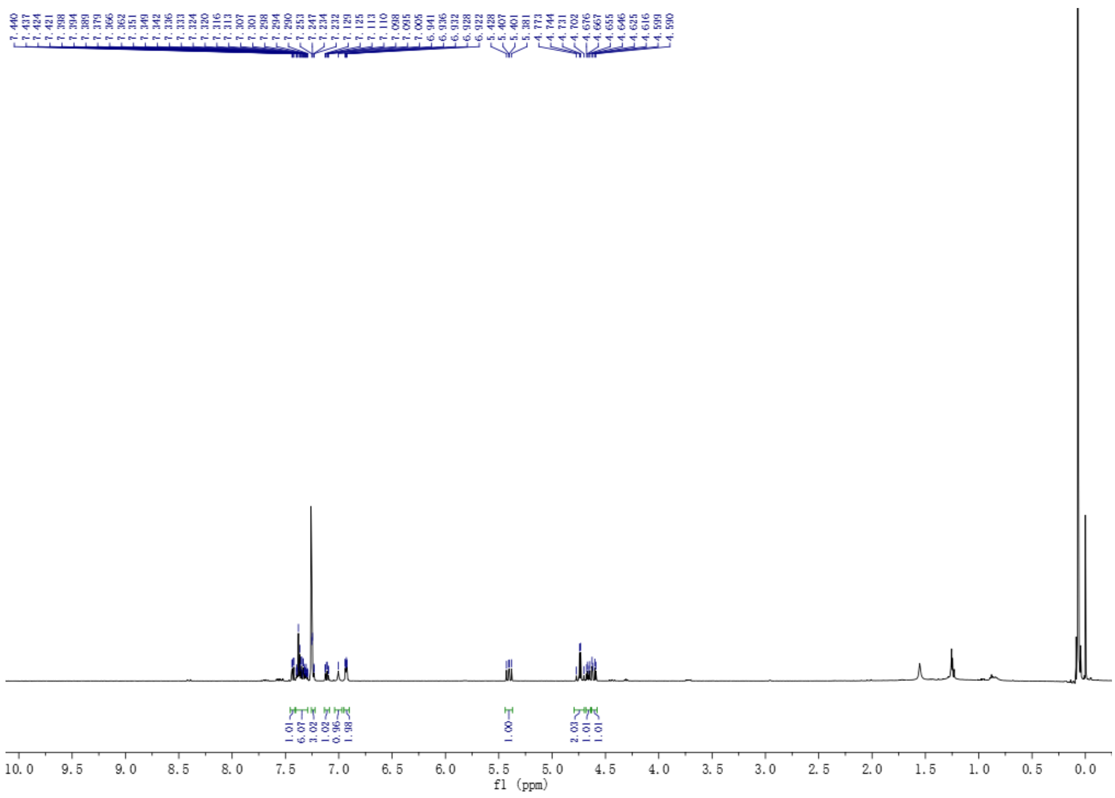

Figure S25. ^{1}H NMR spectrum of **4r**

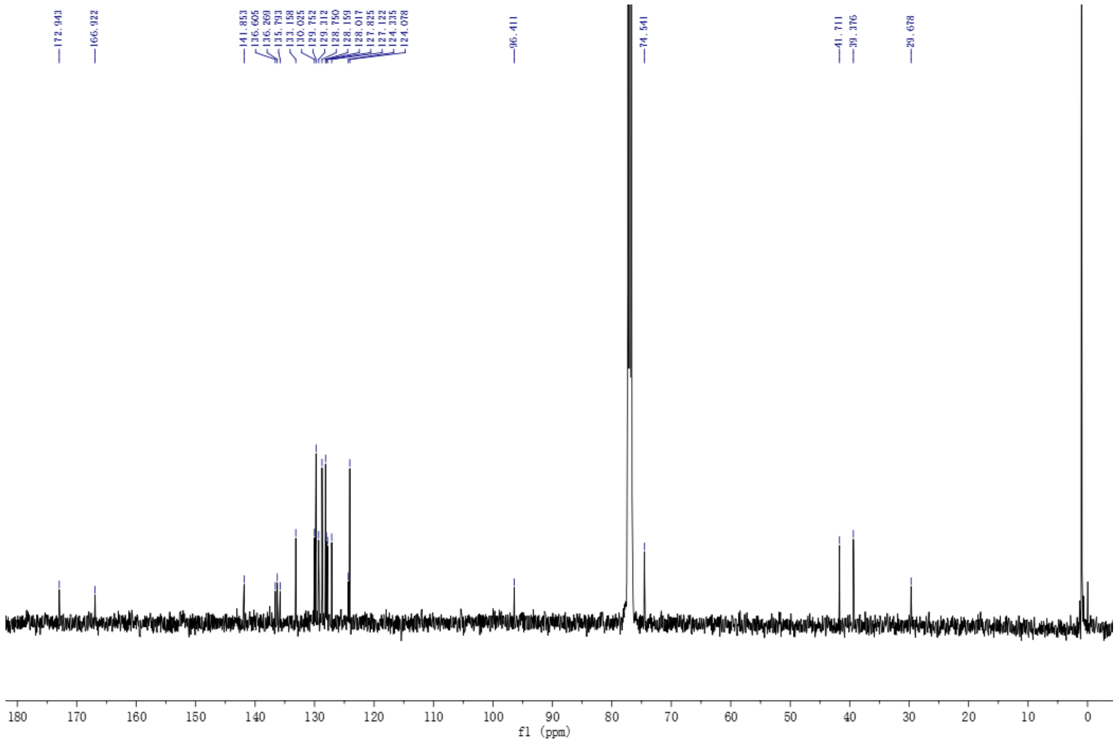

Figure S26. ^{13}C NMR spectrum of **4r**

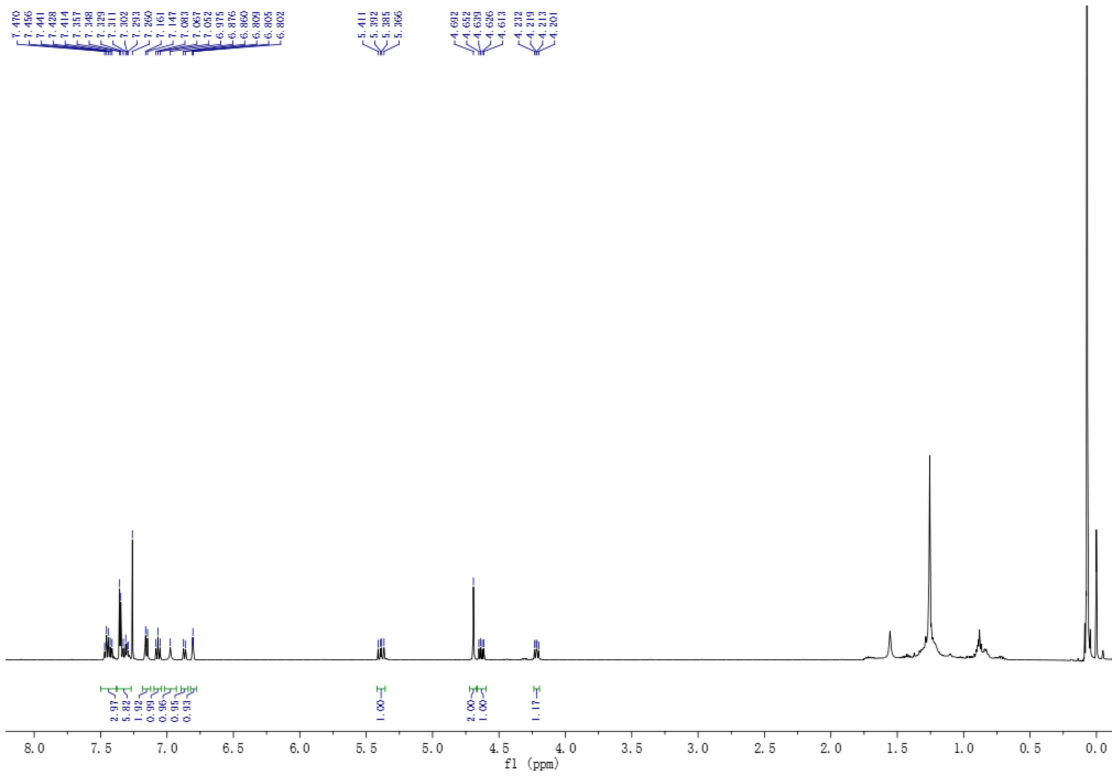

Figure S27. ^{1}H NMR spectrum of **4s**

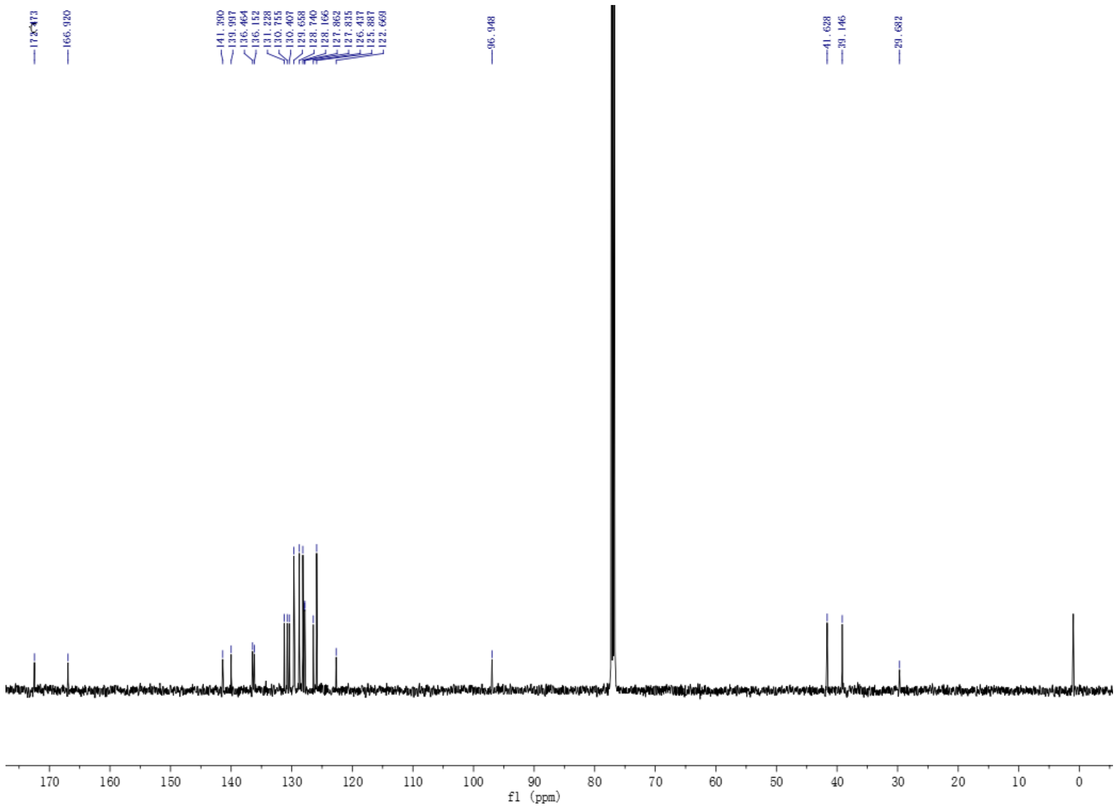

Figure S28. ^{13}C NMR spectrum of **4s**

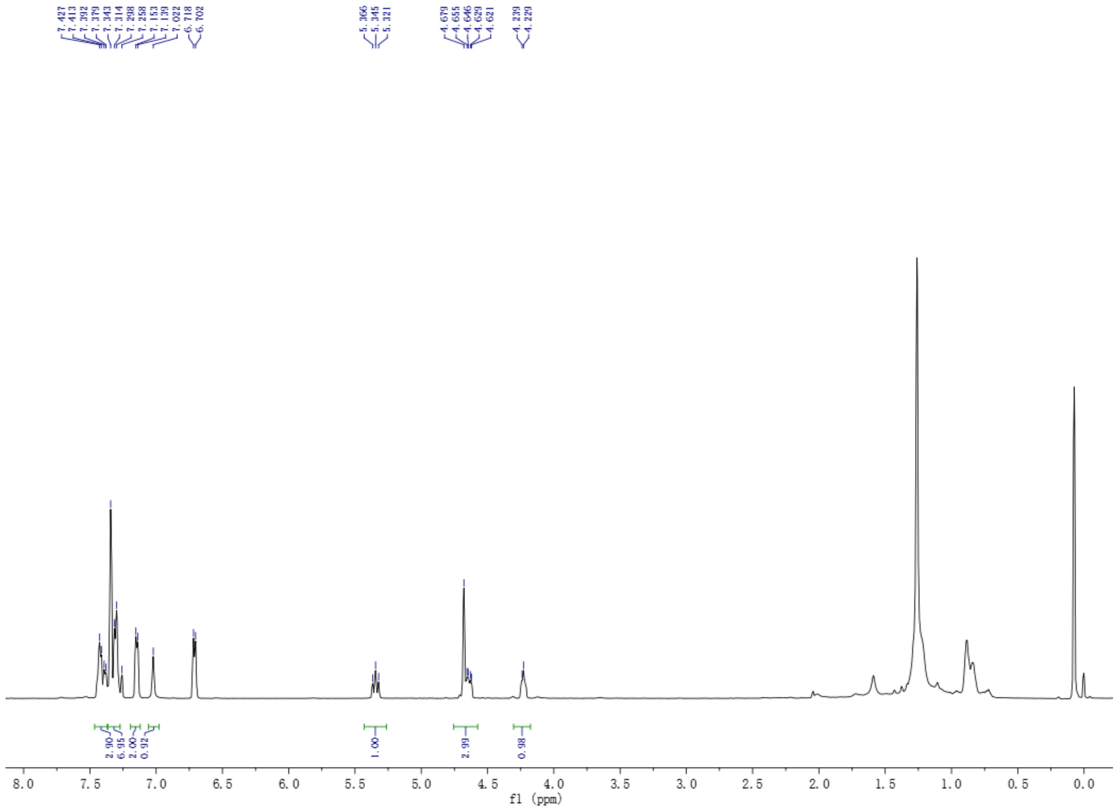

Figure S29. ^{1}H NMR spectrum of **4t**

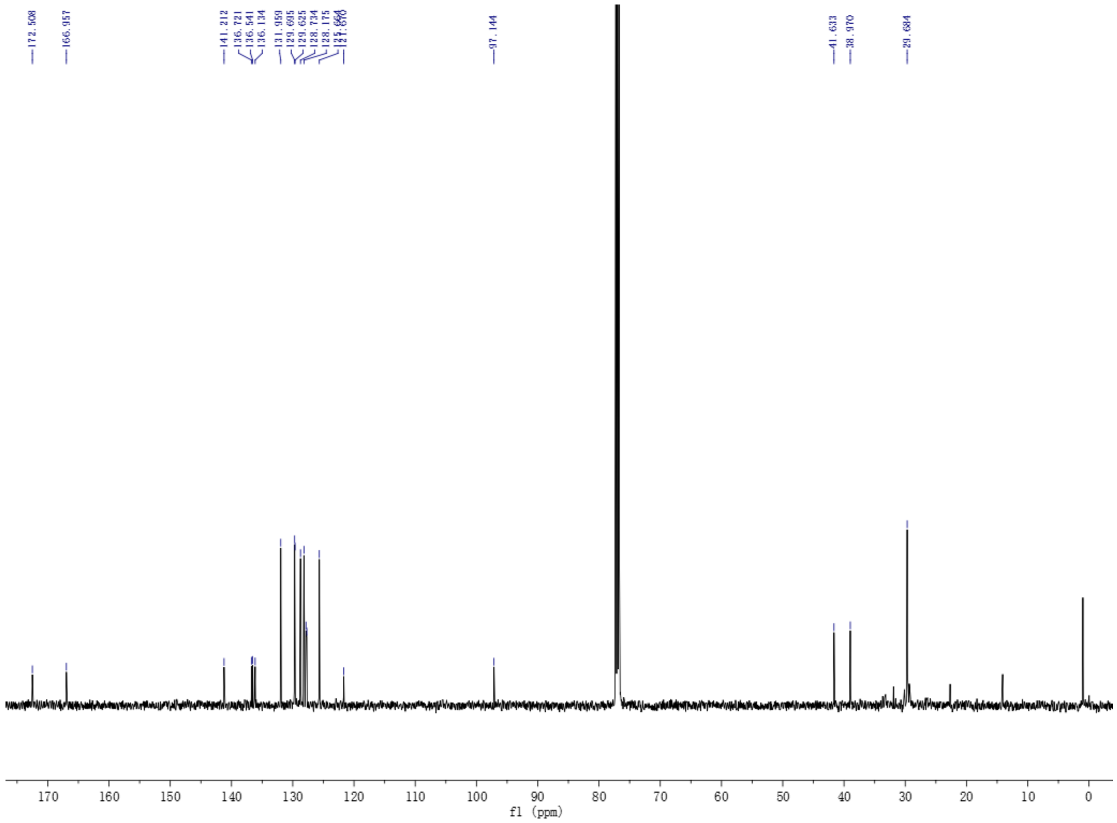

Figure S30. ^{13}C NMR spectrum of **4t**

HPLC trace

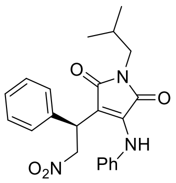

4a

(S) -1-isobutyl-3-(2-nitro-1-phenylethyl)-4-(phenylamino)-1*H*-pyrrole-2,5-dione

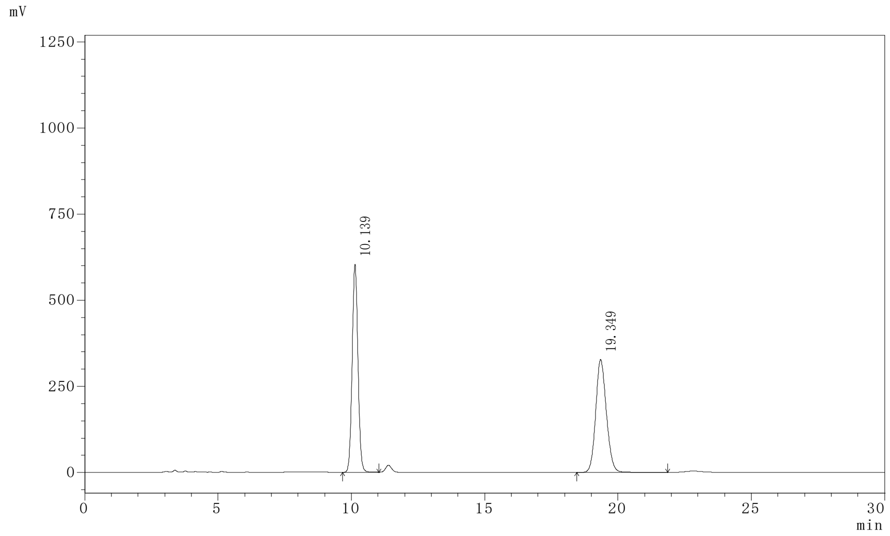

Figure S31. HPLC trace of racemic **4a**

	Retention Time	Area	Height	Area%
1	10.139	9138960	603865	49.893
2	19.349	9178092	327941	50.107
Total		18317052	931805	100.000

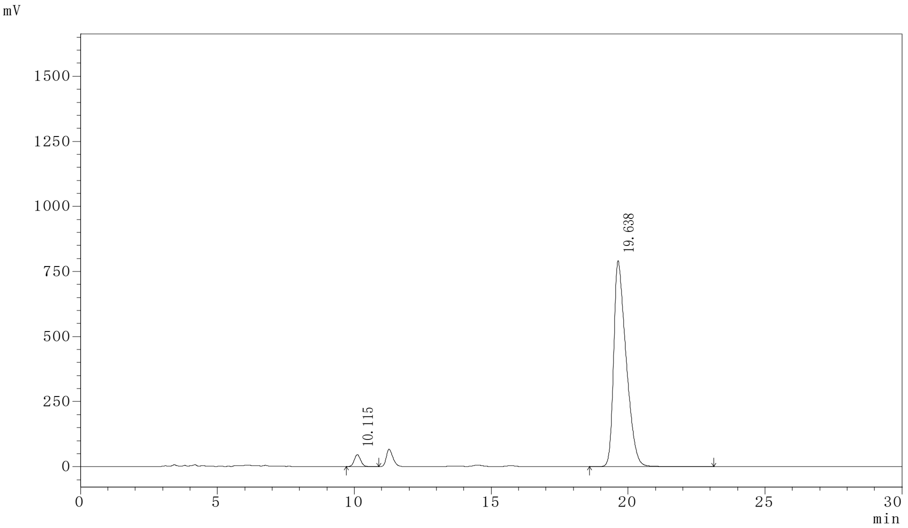

Figure S32. HPLC trace of enantiomeric **4a**

	Retention Time	Area	Height	Area%
1	10.115	759334	46101	2.945
2	19.638	25021650	791063	97.055
total		25780984	837165	100.000

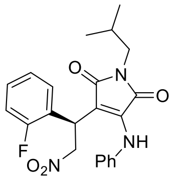

4b

(*S*)-3-(1-(2-fluorophenyl)-2-nitroethyl)-1-isobutyl-4-(phenylamino)-1*H*-pyrrole-2,5-dione

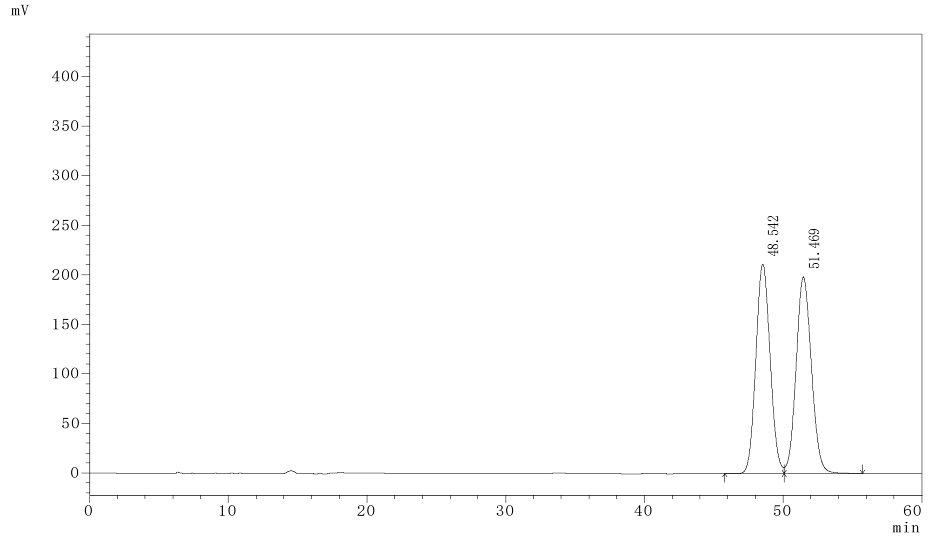

Figure S33. HPLC trace of racemic **4b**

	Retention Time	Area	Height	Area%
1	48.542	15155001	211449	49.808
2	51.469	15271667	198358	50.192
Total		30426669	409807	100.000

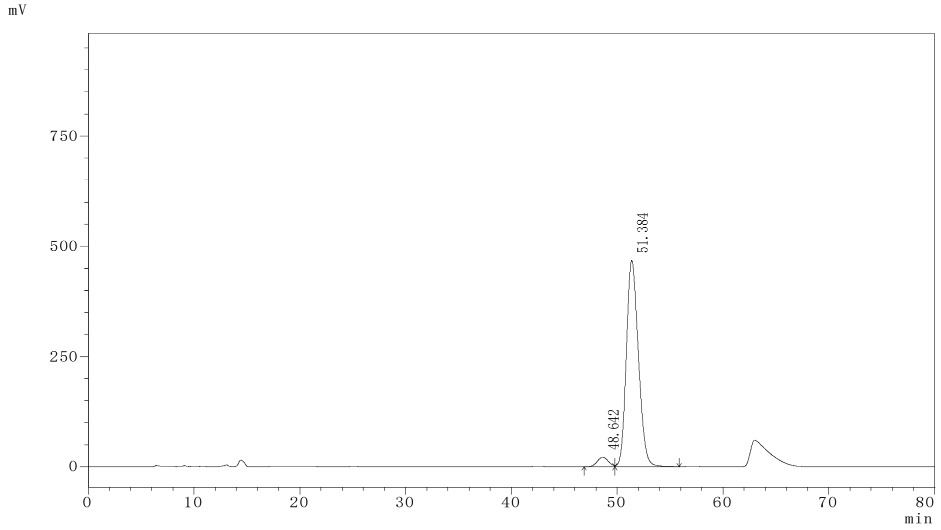

Figure S34. HPLC trace of enantiomeric **4b**

	Retention Time	Area	Height	Area%
1	48.642	1578632	21634	4.093
2	51.384	36989231	467163	95.907
Total		38567864	488797	100.000

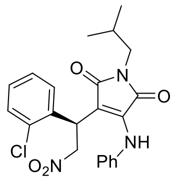

4c

(*S*)-3-(1-(2-chlorophenyl)-2-nitroethyl)-1-isobutyl-4-(phenylamino)-1*H*-pyrrole-2,5-dione

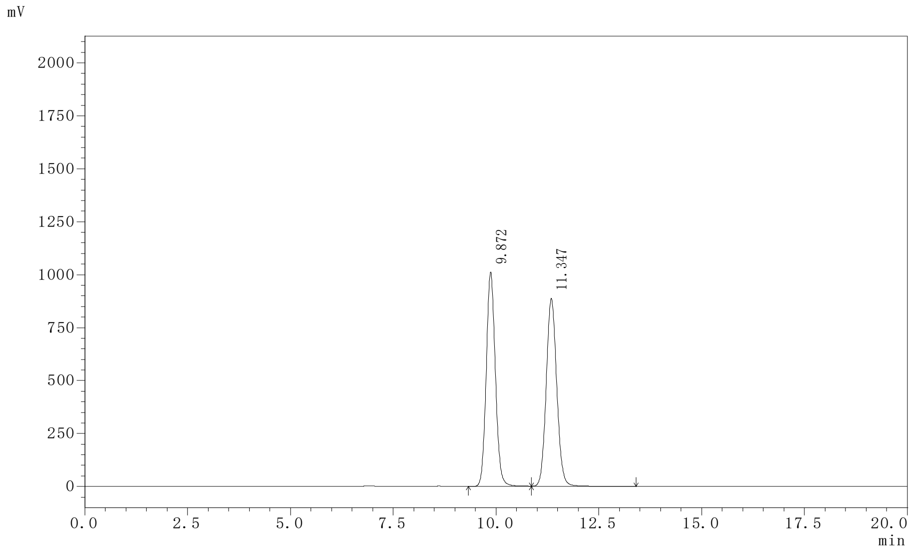

Figure S35. HPLC trace of racemic **4c**

	Retention Time	Area	Height	Area%
1	9.872	15655871	1011725	49.658
2	11.347	15871482	887786	50.342
Total		31527353	1899511	100.000

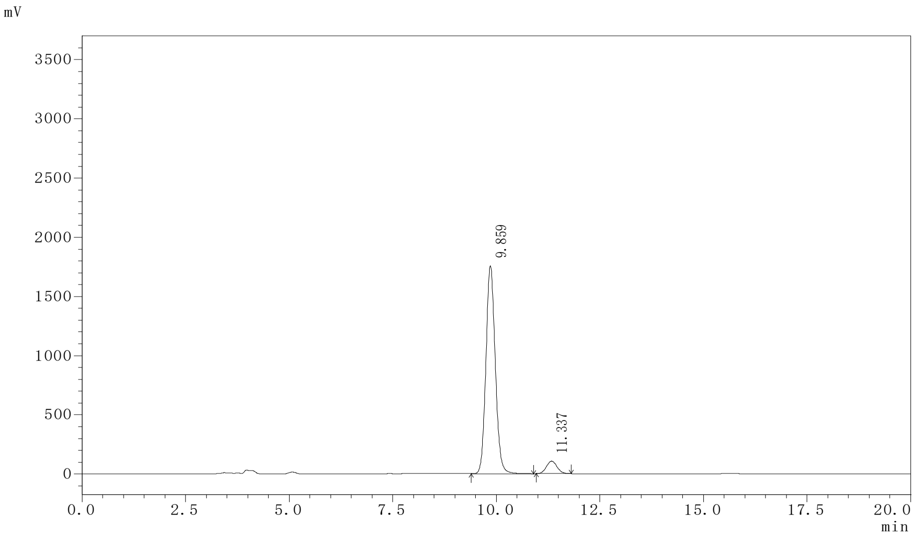

Figure S36. HPLC trace of enantiomeric **4c**

	Retention Time	Area	Height	Area%

1	9.859	27816465	1761326	93.590
2	11.337	1905188	106081	6.410
Total		29721652	1867407	100.000

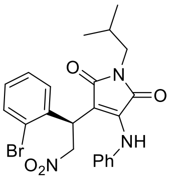

4d

(*S*)-3-(1-(2-bromophenyl)-2-nitroethyl)-1-isobutyl-4-(phenylamino)-1*H*-pyrrole-2,5-dione

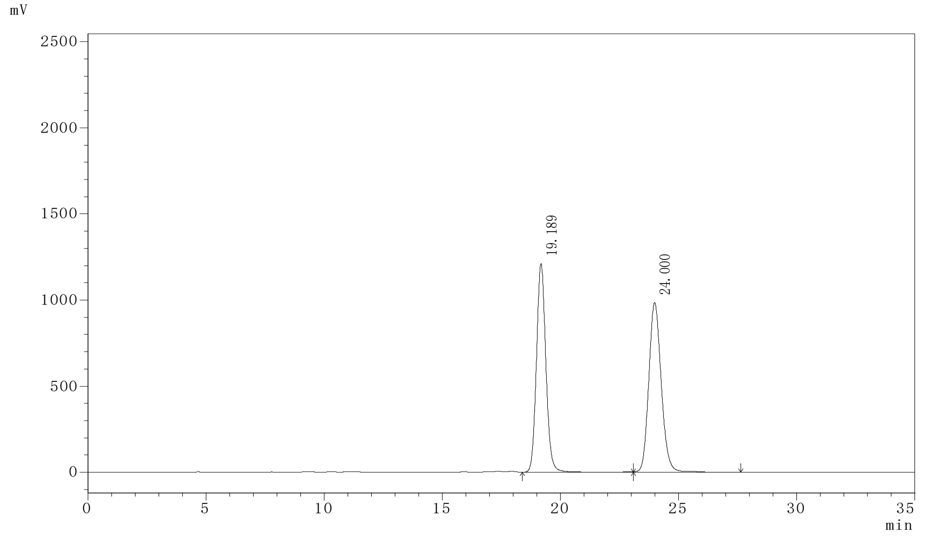

Figure S37. HPLC trace of racemic **4d**

	Retention Time	Area	Height	Area%
1	19.189	33636687	1211148	48.189
2	24.000	36164614	985199	51.811
Total		69801301	2196347	100.000

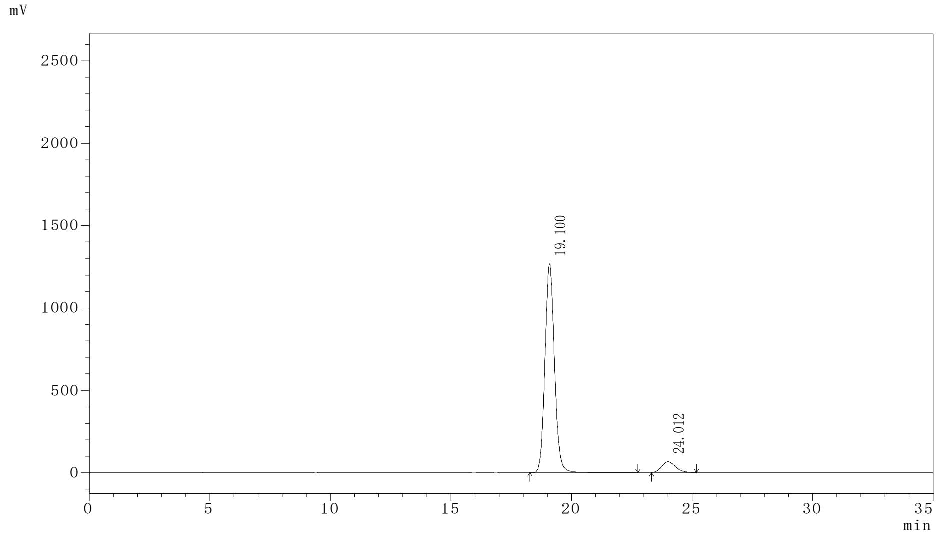

Figure S38. HPLC trace of enantiomeric **4d**

	Retention Time	Area	Height	Area%
1	19.100	34784311	1267197	93.031
2	24.012	2605764	65406	6.969
Total		37390075	1332603	100.000

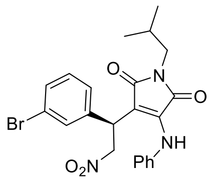

4e

(*S*)-3-(1-(3-bromophenyl)-2-nitroethyl)-1-isobutyl-4-(phenylamino)-1*H*-pyrrole-2,5-dione

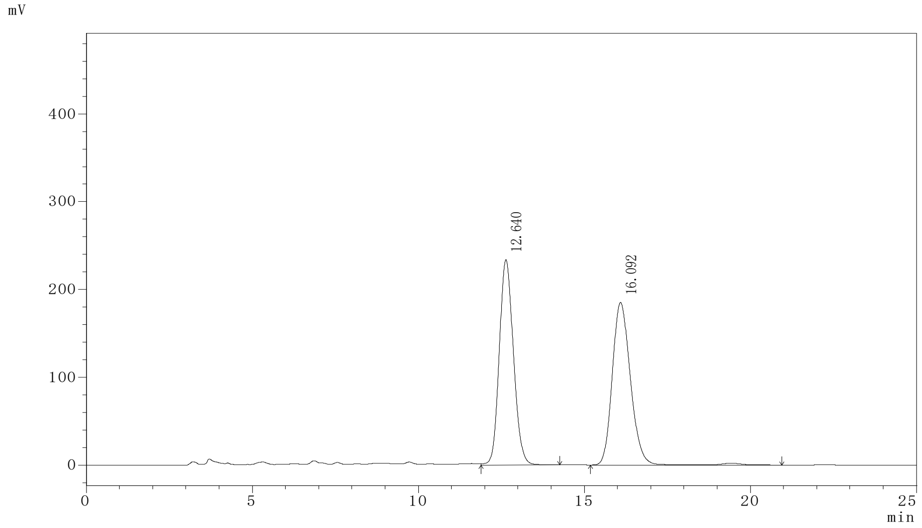

Figure S39. HPLC trace of racemic **4e**

	Retention Time	Area	Height	Area%
1	12.640	6934477	233645	49.334
2	16.092	7121822	185030	50.666
Total		14056300	418675	100.000

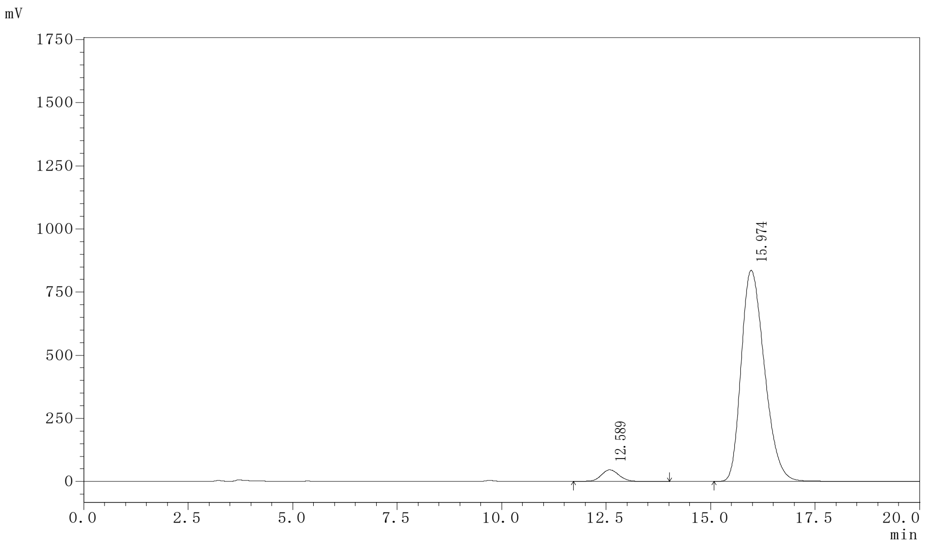

Figure S40. HPLC trace of enantiomeric **4e**

	Retention Time	Area	Height	Area%
1	12.589	1380644	46246	4.086
2	15.974	32409313	836391	95.914
Total		33789957	882637	100.000

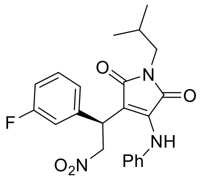

4f

(*S*)-3-(1-(3-fluorophenyl)-2-nitroethyl)-1-isobutyl-4-(phenylamino)-1*H*-pyrrole-2,5-dione

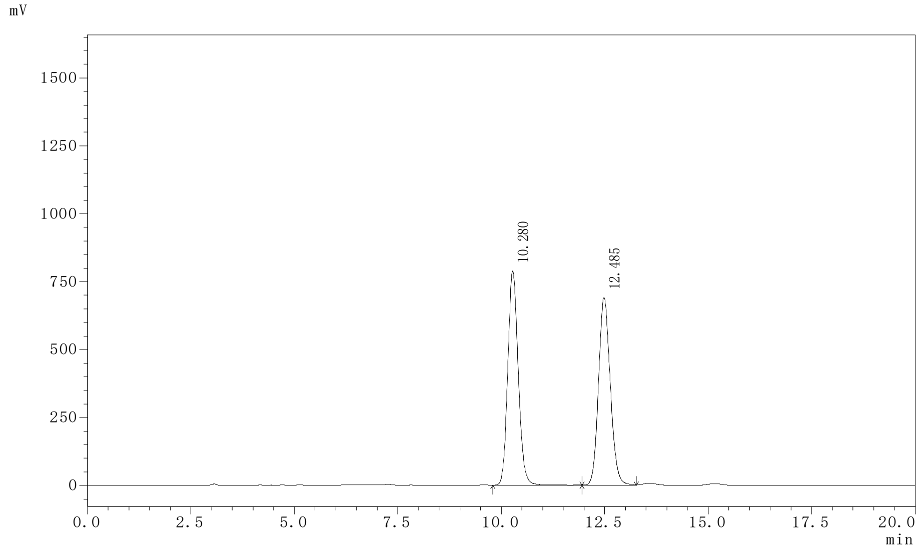

Figure S41. HPLC trace of racemic **4f**

	Retention Time	Area	Height	Area%
1	10.280	13934923	789206	50.230

2	12.485	13807159	692392	49.770
Total		27742082	1481599	100.000

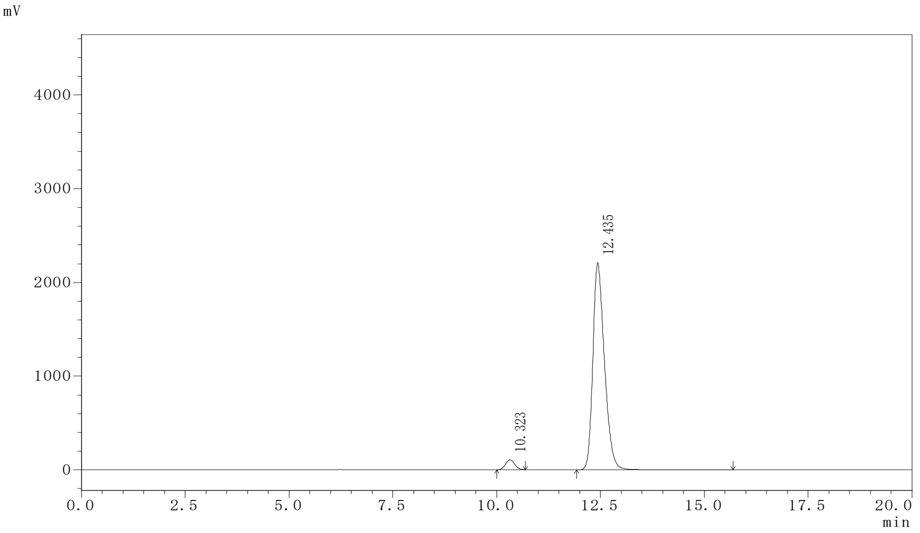

Figure S42. HPLC trace of enantiomeric **4f**

	Retention Time	Area	Height	Area%
1	10.323	1633344	105011	3.657
2	12.435	43031262	2210246	96.343
Total		44664606	2315257	100.000

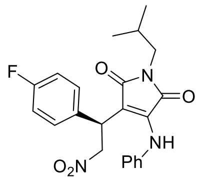

4g

(*S*)-3-(1-(4-fluorophenyl)-2-nitroethyl)-1-isobutyl-4-(phenylamino)-1*H*-pyrrole-2,5-dione

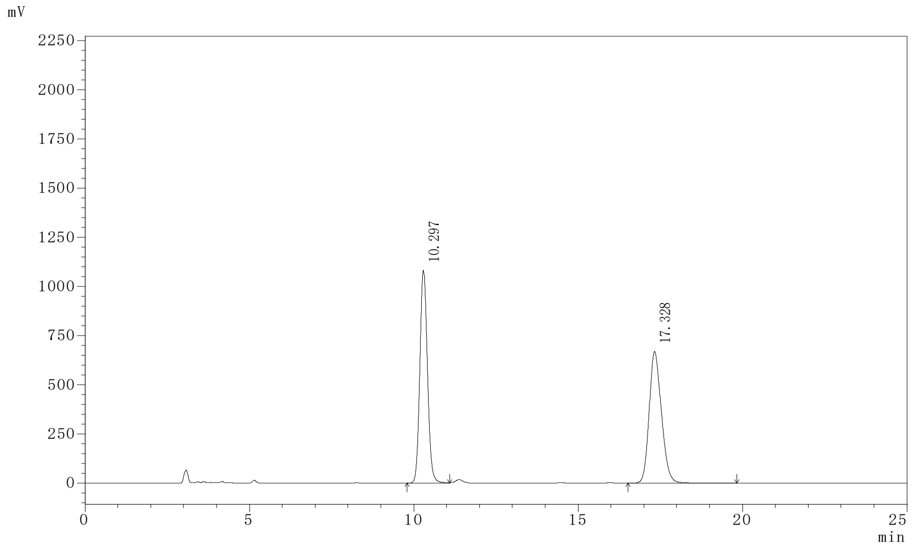

Figure S43. HPLC trace of racemic **4g**

	Retention Time	Area	Height	Area%
1	10.297	17355813	1080958	49.932
2	17.328	17403111	669000	50.068
Total		34758924	1749958	100.000

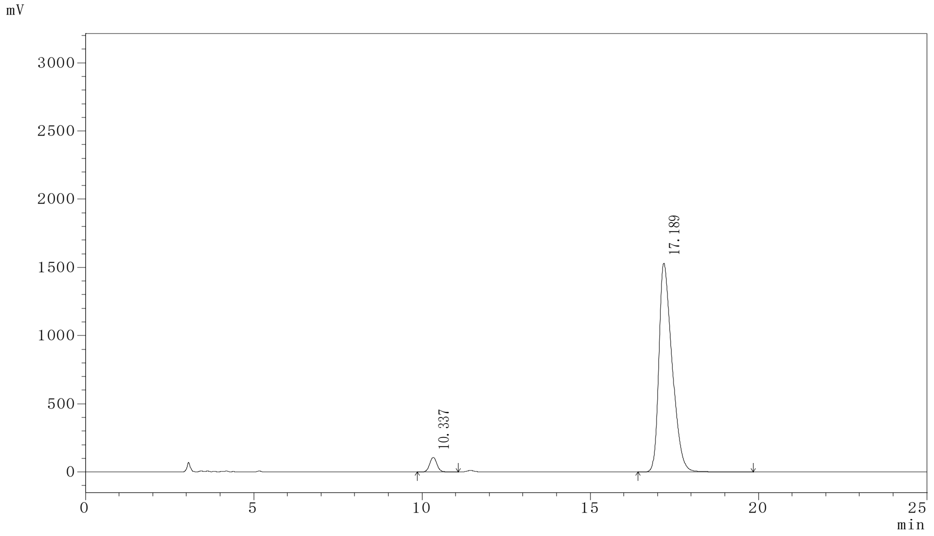

Figure S44. HPLC trace of enantiomeric **4g**

	Retention Time	Area	Height	Area%
1	10.337	1628391	106789	3.769
2	17.189	41575627	1529873	96.231
Total		43204018	1636662	100.000

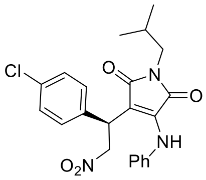

4h

(S)-3-(1-(4-Chlorophenyl)-2-nitroethyl)-1-isobutyl-4-(phenylamino)-1H-pyrrole-2,5-dione

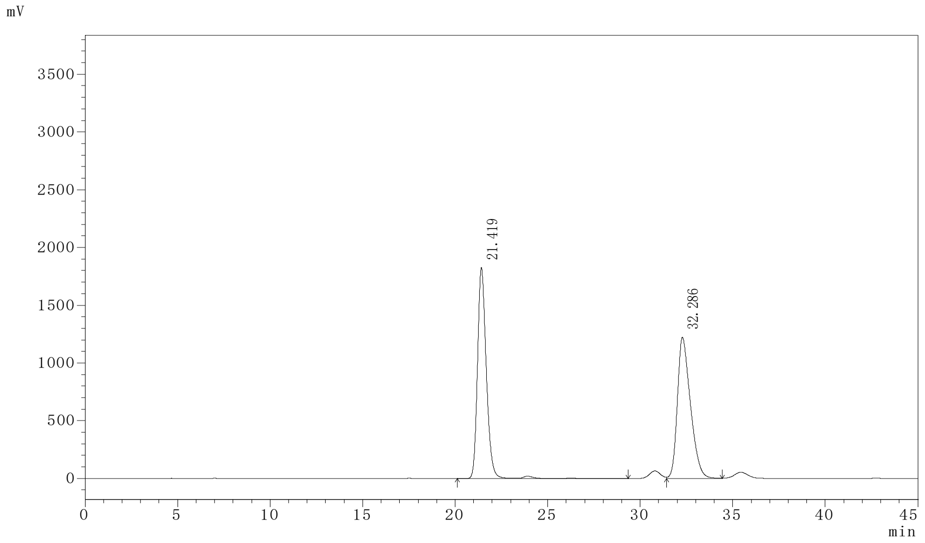

Figure S45. HPLC trace of racemic **4h**

	Retention Time	Area	Height	Area%
1	21.419	60754071	1826744	50.318
2	32.286	59986437	1224105	49.682
Total		120740509	3050849	100.000

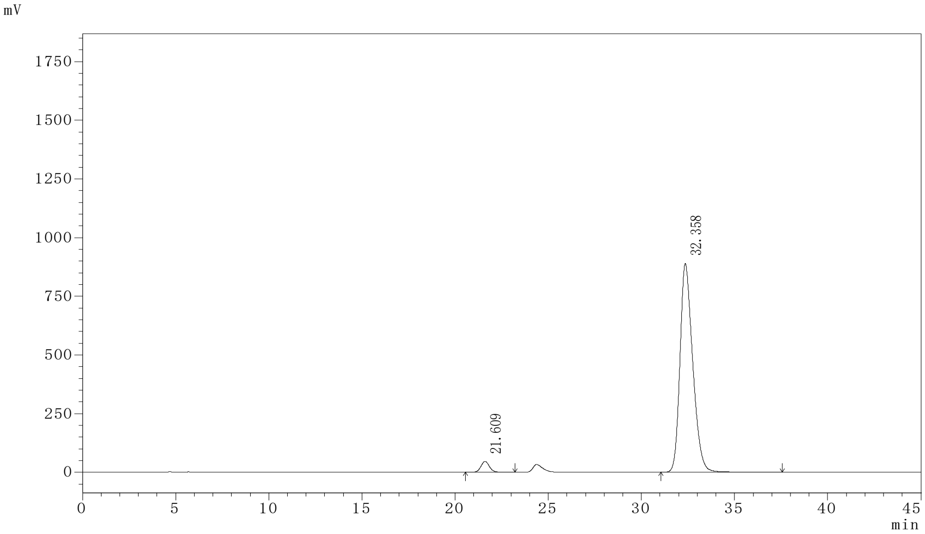

Figure S46. HPLC trace of enantiomeric **4h**

	Retention Time	Area	Height	Area%
1	21.609	1466990	46517	3.357

2	32.358	42226262	889083	96.643
Total		43693252	935600	100.000

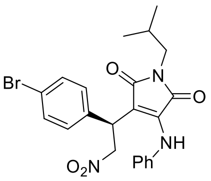

4i

(*S*)-3-(1-(4-bromophenyl)-2-nitroethyl)-1-isobutyl-4-(phenylamino)-1*H*-pyrrole-2,5-dione

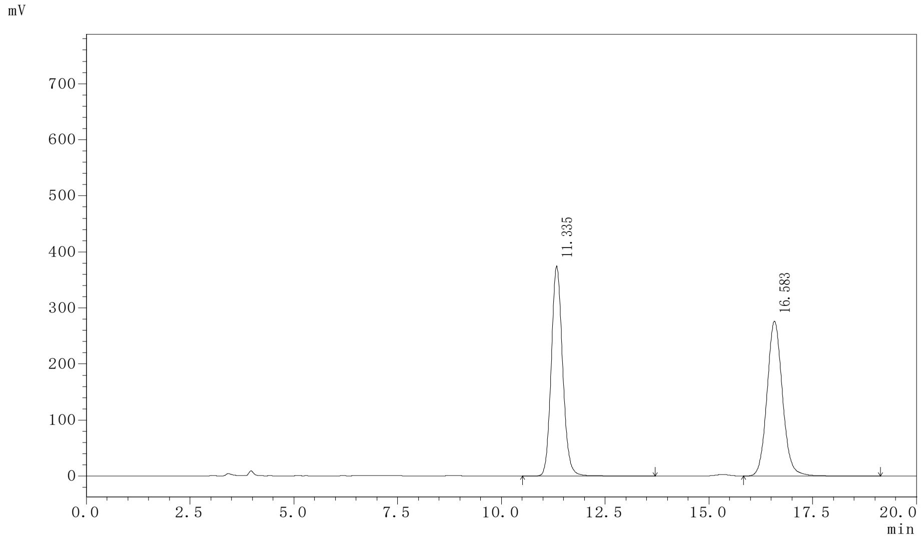

Figure S47. HPLC trace of racemic **4i**

	Retention Time	Area	Height	Area%
1	11.335	7133500	374943	49.951
2	16.583	7147600	276358	50.049
Total		14281100	651302	100.000

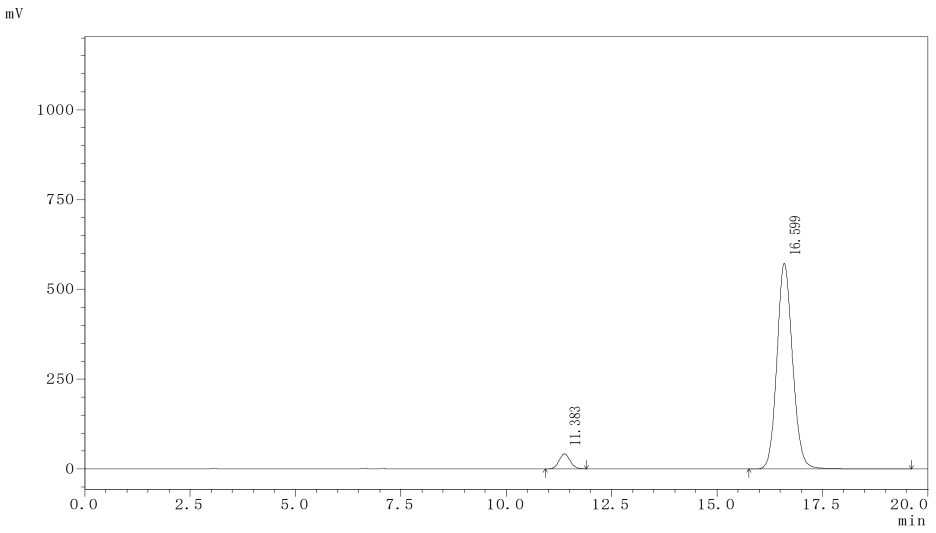

Figure S48. HPLC trace of enantiomeric **4i**

	Retention Time	Area	Height	Area%
1	11.383	771017	41966	4.970
2	16.599	14741462	572543	95.030
Total		15512478	614509	100.000

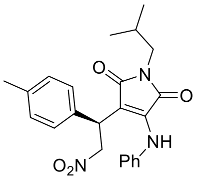

4j

(*S*)-1-isobutyl-3-(2-nitro-1-(p-tolyl)ethyl)-4-(phenylamino)-1*H*-pyrrole-2,5-dione

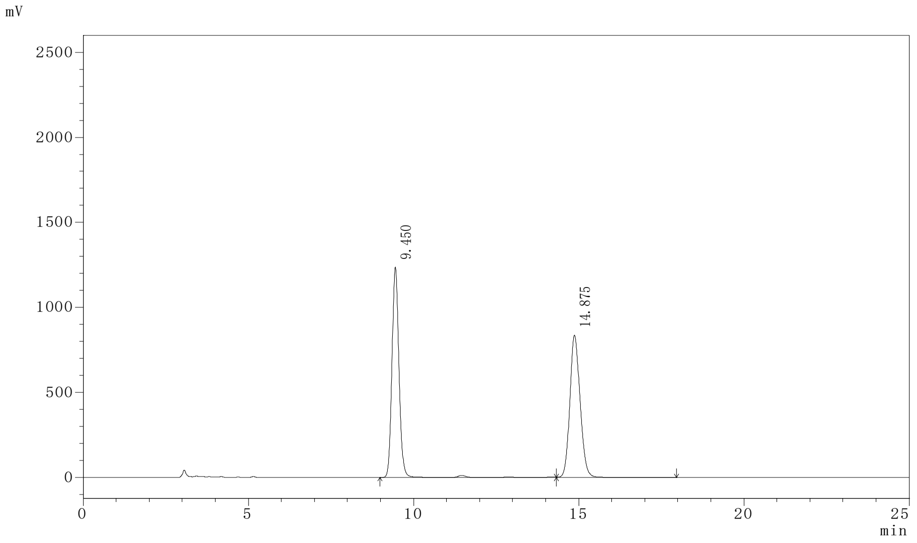

Figure S49. HPLC trace of racemic **4j**

	Retention Time	Area	Height	Area%
1	9.450	18510129	1236848	50.506
2	14.875	18139311	837292	49.494

Total		36649440	2074140	100.000

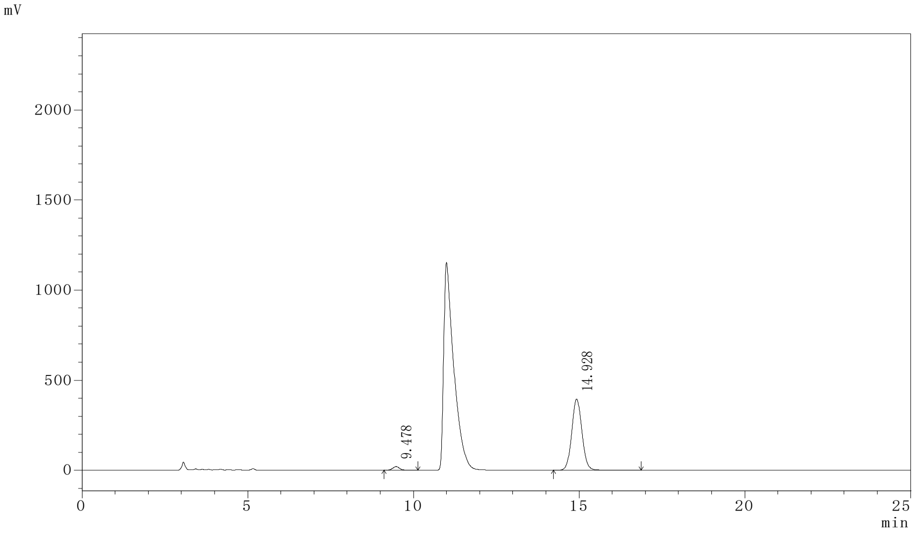

Figure S50. HPLC trace of enantiomeric **4j**

	Retention Time	Area	Height	Area%
1	9.478	294495	20289	3.358
2	14.928	8476652	395554	96.642
Total		8771147	415843	100.000

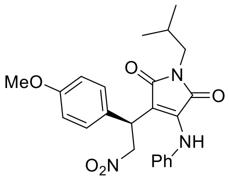

4k

(*S*)-1-isobutyl-3-(1-(4-methoxyphenyl)-2-nitroethyl)-4-(phenylamino)-1*H*-pyrrole-2,5-dion
e

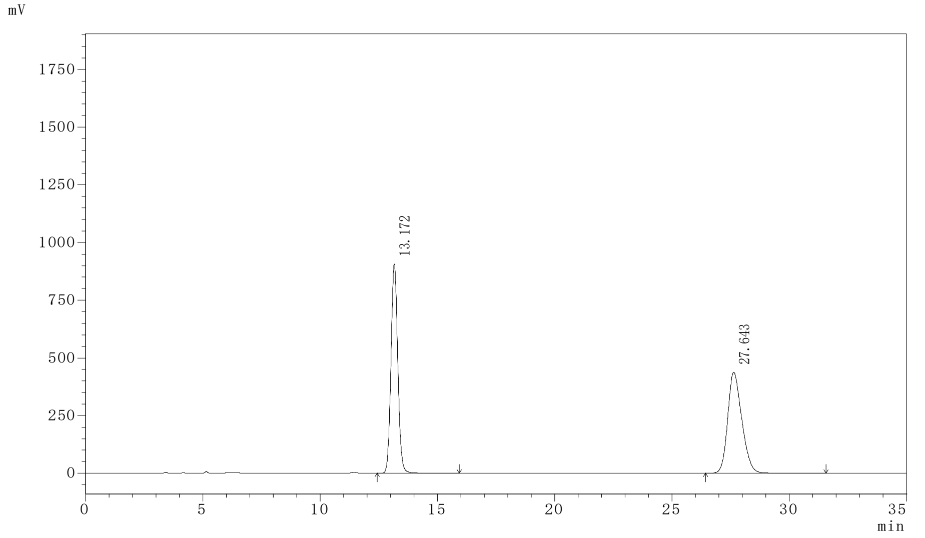

Figure S51. HPLC trace of racemic **4k**

	Retention Time	Area	Height	Area%
1	13.172	18294983	906257	49.966
2	27.643	18320176	438026	50.034
Total		36615159	1344283	100.000

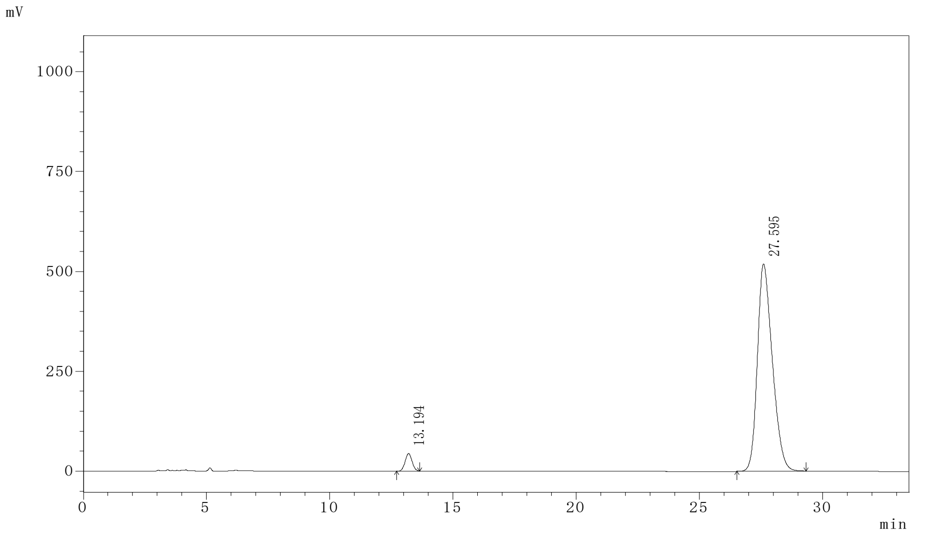

Figure S52. HPLC trace of enantiomeric **4k**

	Retention Time	Area	Height	Area%
1	13.194	863962	44000	3.793
2	27.595	21912863	519333	96.207
Total		22776825	563333	100.000

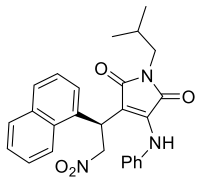

4l

(S)-1-isobutyl-3-(1-(naphthalen-2-yl)-2-nitroethyl)-4-(phenylamino)-1H-pyrrole-2,5-dione

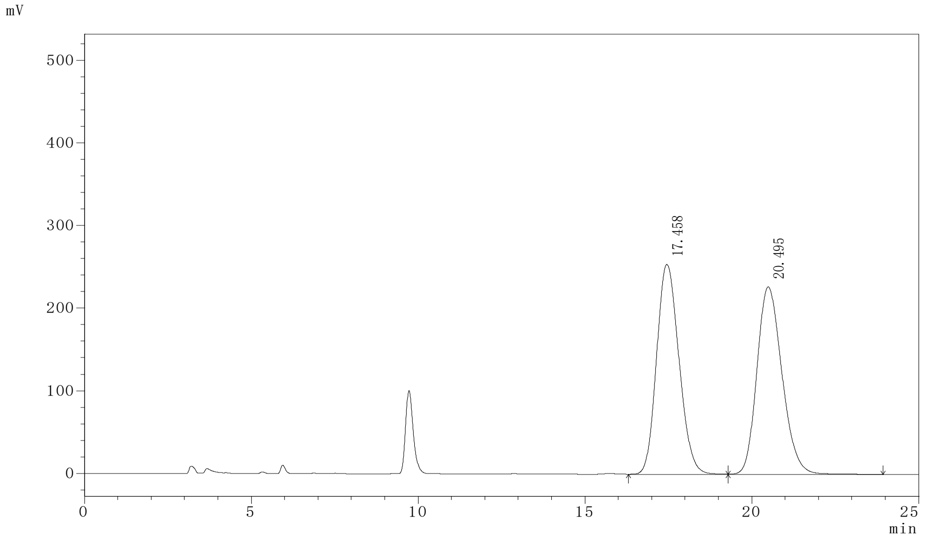

Figure S53. HPLC trace of racemic **4l**

	Retention Time	Area	Height	Area%
1	17.458	11874462	254203	49.982
2	20.495	11883083	227158	50.018
Total		23757545	481360	100.000

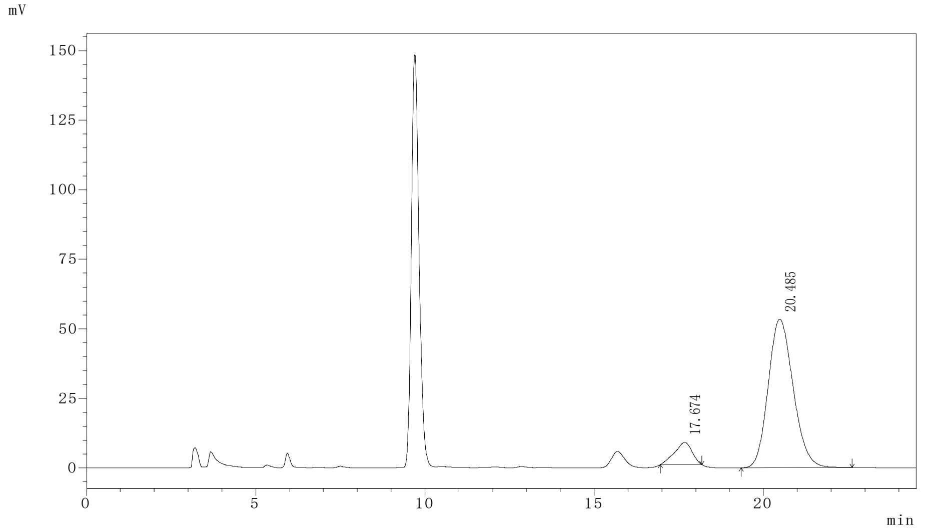

Figure S54. HPLC trace of enantiomeric **4l**

	Retention Time	Area	Height	Area%
1	17.674	288976	8013	9.549
2	20.485	2737292	53380	90.451
Total		3026268	61392	100.000

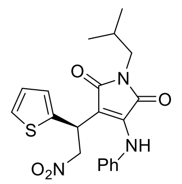

4m

(*S*)-1-isobutyl-3-(2-nitro-1-(thiophen-2-yl)ethyl)-4-(phenylamino)-1*H*-pyrrole-2,5-dione

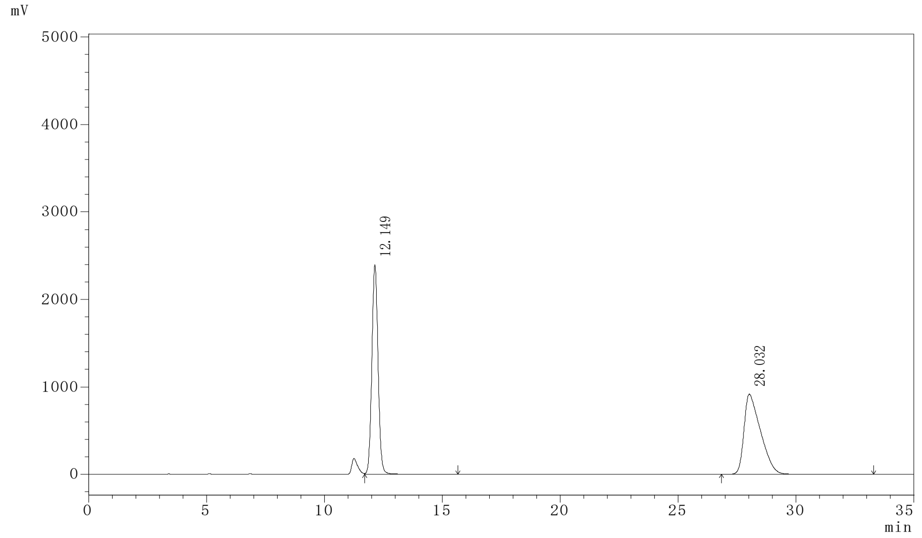

Figure S55. HPLC trace of racemic **4m**

	Retention Time	Area	Height	Area%
1	12.149	43993359	2396140	49.534
2	28.032	44820465	917713	50.466
Total		88813824	3313853	100.000

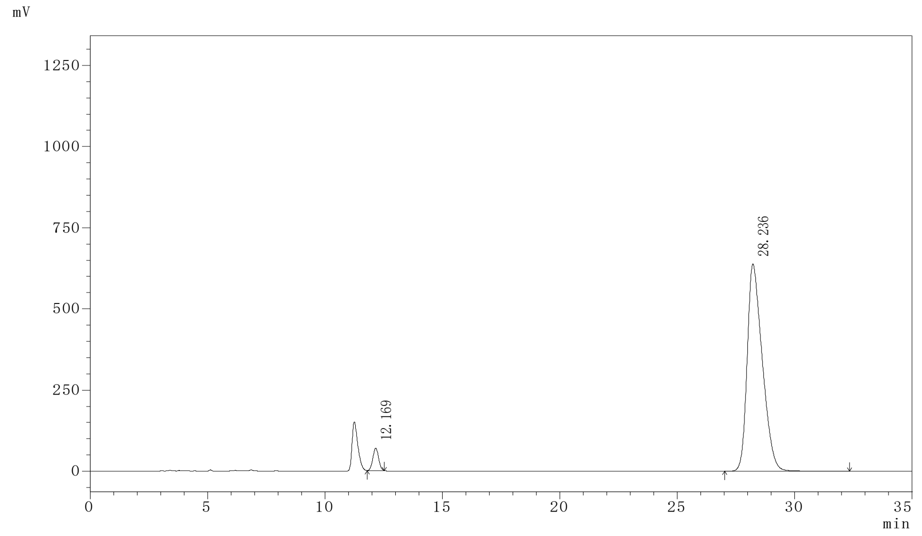

Figure S56. HPLC trace of enantiomeric **4m**

	Retention Time	Area	Height	Area%
1	12.169	1177306	68735	3.958
2	28.236	28571204	638419	96.042
Total		29748510	707155	100.000

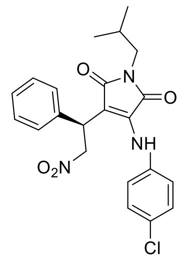

4n

S)-3-((4-chlorophenyl)amino)-1-isobutyl-4-(2-nitro-1-phenylethyl)-1*H*-pyrrole-2,5-dione

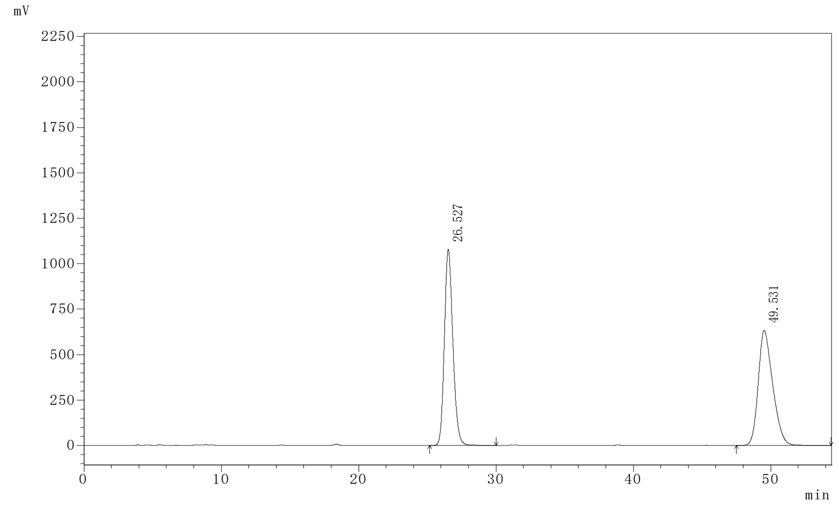

Figure S57. HPLC trace of racemic **4n**

	Retention Time	Area	Height	Area%
1	26.527	46476300	1079072	49.999
2	49.531	46478002	633583	50.001
Total		92954302	1712654	100.000

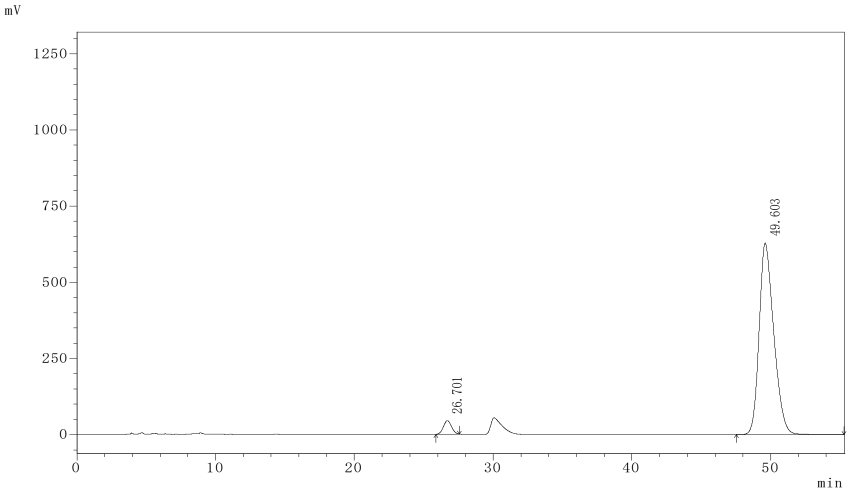

Figure S58. HPLC trace of enantiomeric **4n**

	Retention Time	Area	Height	Area%
1	26.701	1821309	44453	3.797
2	49.603	46146238	628641	96.203

Total		47967547	673094	100.000

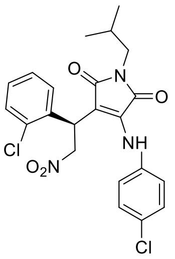

4o

(*S*)-3-((4-chlorophenyl)amino)-1-isobutyl-4-(1-(2-chlorophenyl)-2-nitroethyl)-1*H*-pyrrole-2,5-dione

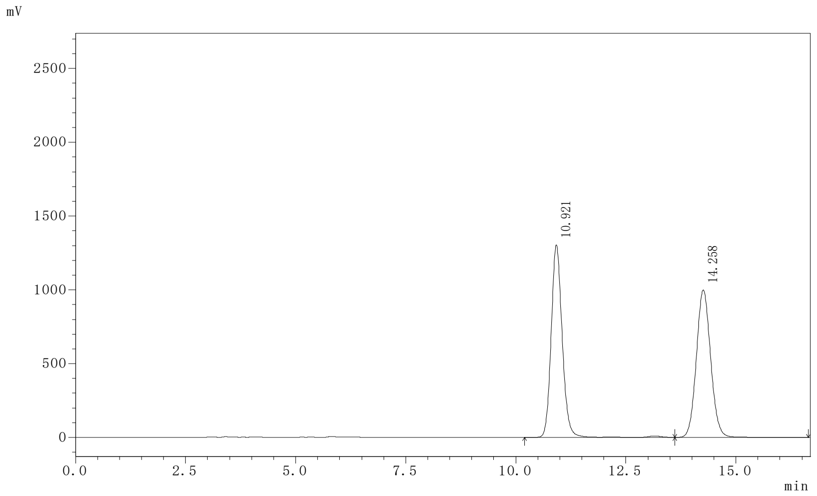

Figure S59. HPLC trace of racemic **4o**

	Retention Time	Area	Height	Area%
1	10.921	22915691	1303387	50.365
2	14.258	22583928	1000139	49.635
Total		45499620	2303526	100.000

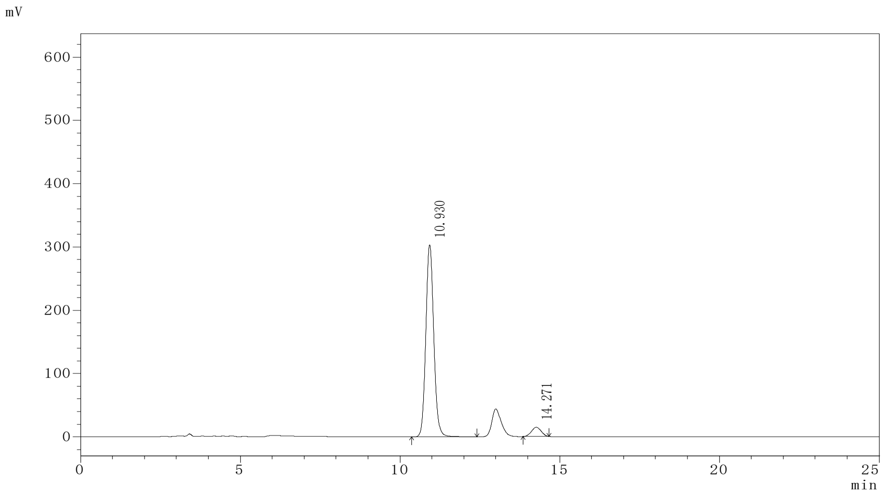

Figure S60. HPLC trace of enantiomeric **4o**

	Retention Time	Area	Height	Area%
1	10.930	5238139	303049	94.410
2	14.271	310162	14672	5.590
Total		5548302	317720	100.000

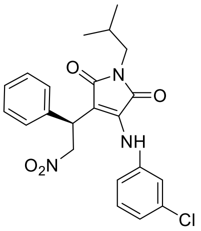

4p

(*S*)-3-((3-chlorophenyl)amino)-1-isobutyl-4-(2-nitro-1-phenylethyl)-1*H*-pyrrole-2,5-dione

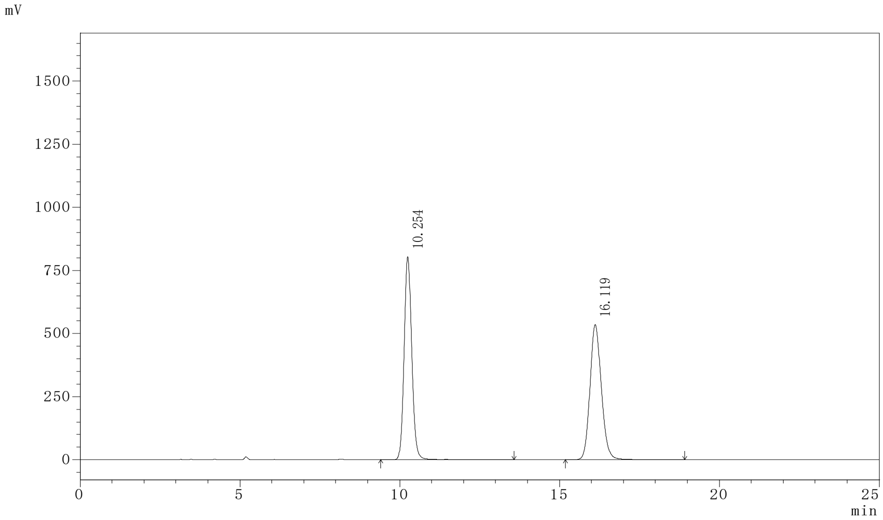

Figure S61. HPLC trace of racemic **4p**

	Retention Time	Area	Height	Area%
1	10.254	13397123	804118	50.164
2	16.119	13309657	535510	49.836
Total		26706781	1339628	100.000

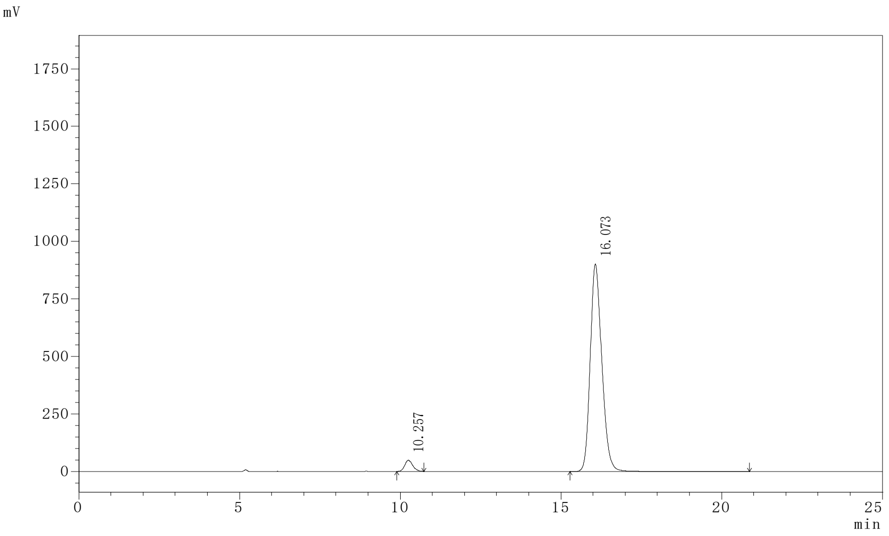

Figure S62. HPLC trace of enantiomeric **4p**

	Retention Time	Area	Height	Area%
1	10.257	841474	48423	3.574
2	16.073	22704873	901729	96.426
Total		23546346	950152	100.000

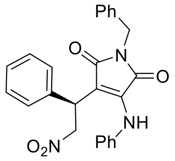

4q

(*S*)-1-benzyl-3-(2-nitro-1-phenylethyl)-4-(phenylamino)-1*H*-pyrrole-2,5-dione

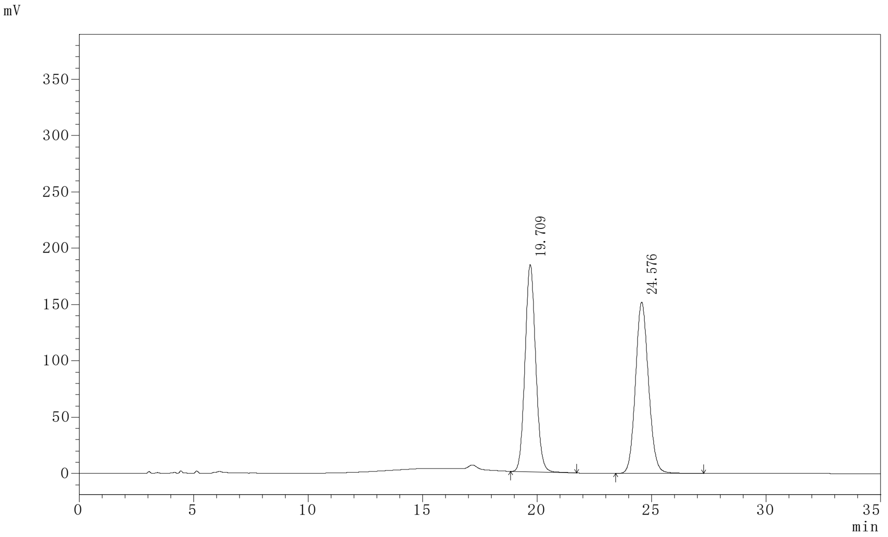

Figure S63. HPLC trace of racemic **4q**

	Retention Time	Area	Height	Area%
1	19.709	5974205	184053	49.835

2	24.576	6013836	152156	50.165
Total		11988041	336209	100.000

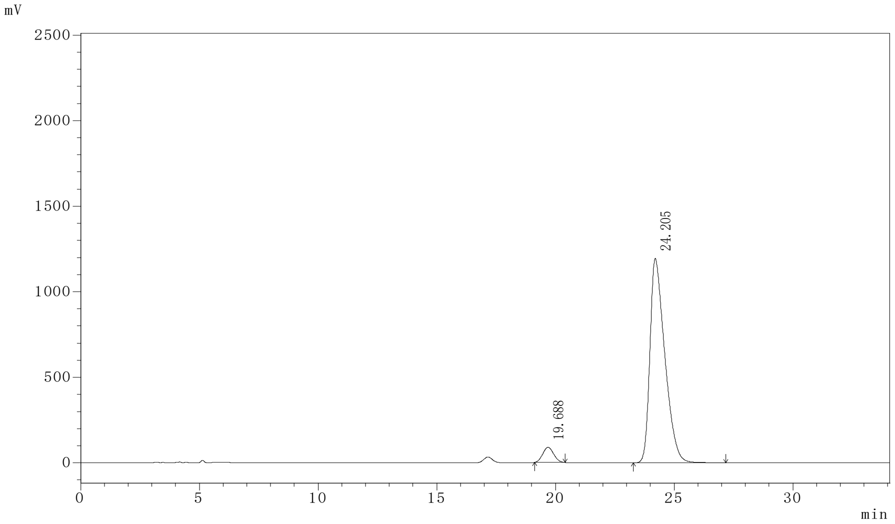

Figure S64. HPLC trace of enantiomeric **4q**

	Retention Time	Area	Height	Area%
1	19.688	2848800	88951	5.189
2	24.205	52049596	1194382	94.811
Total		54898396	1283333	100.000

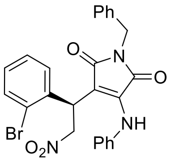

4r

(*S*)-1-benzyl-3-(1-(2-bromophenyl)-2-nitroethyl)-4-(phenylamino)-1*H*-pyrrole-2,5-dione

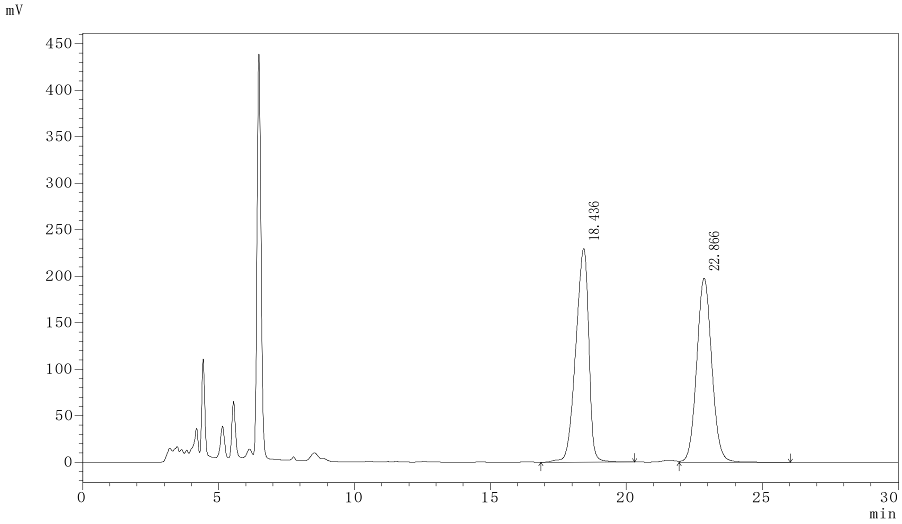

Figure S65. HPLC trace of racemic **4r**

	Retention Time	Area	Height	Area%

1	18.436	7668575	229755	50.145
2	22.866	7624271	197997	49.855
Total		15292846	427752	100.000

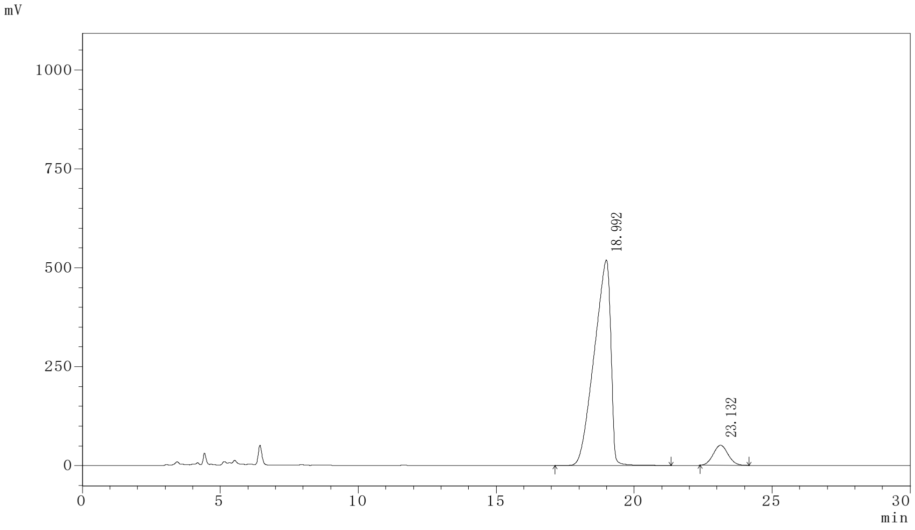

Figure S66. HPLC trace of enantiomeric **4r**

	Retention Time	Area	Height	Area%
1	18.992	21619995	519320	91.955
2	23.132	1891485	50675	8.045
Total		23511481	569994	100.000

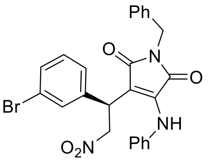

4s

(*S*)-1-benzyl-3-(1-(3-bromophenyl)-2-nitroethyl)-4-(phenylamino)-1*H*-pyrrole-2,5-dione

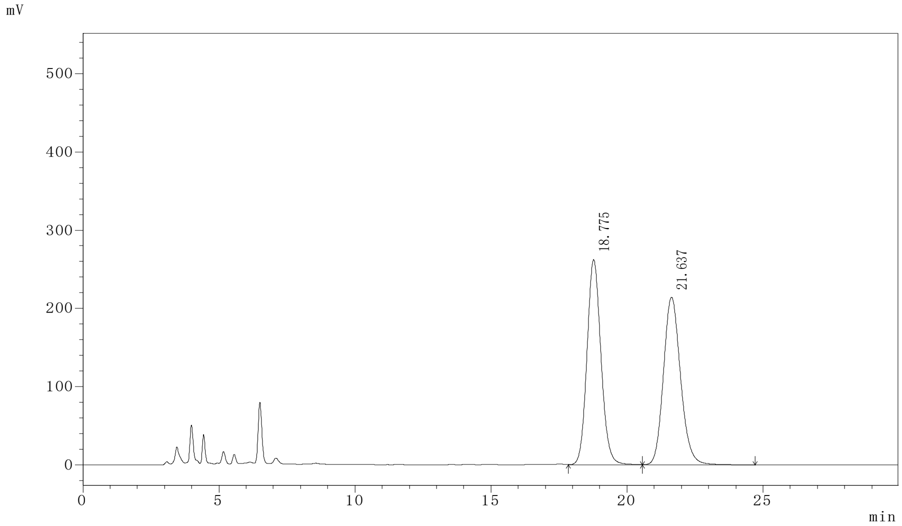

Figure S67. HPLC trace of racemic **4s**

	Retention Time	Area	Height	Area%
1	18.775	9380637	262261	50.014
2	21.637	9375375	214300	49.986
Total		18756012	476560	100.000

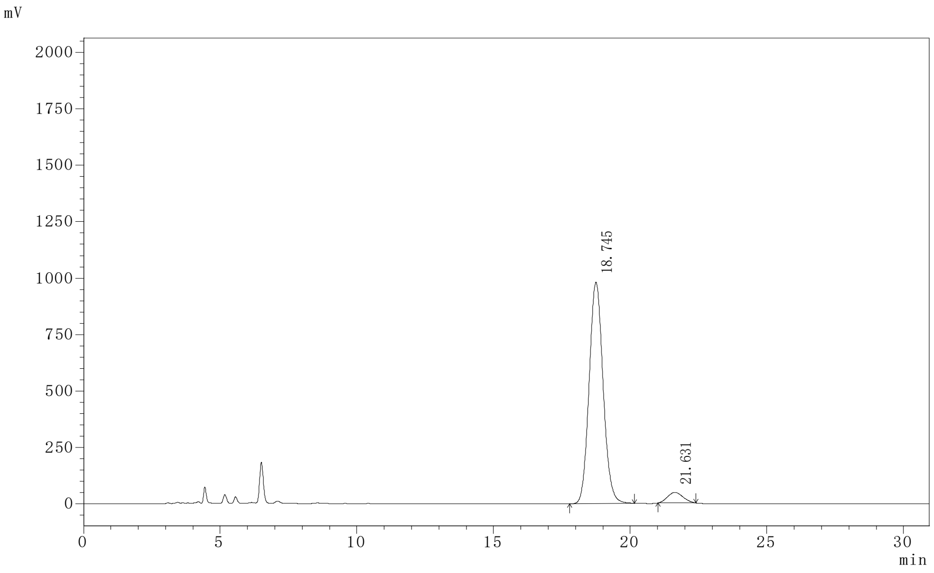

Figure S68. HPLC trace of enantiomeric **4s**

	Retention Time	Area	Height	Area%
1	18.745	34846159	980643	95.017
2	21.631	1827472	46512	4.983
Total		36673631	1027155	100.000

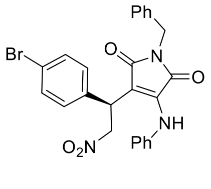

4t

(*S*)-1-benzyl-3-(1-(4-bromophenyl)-2-nitroethyl)-4-(phenylamino)-1*H*-pyrrole-2,5-dione

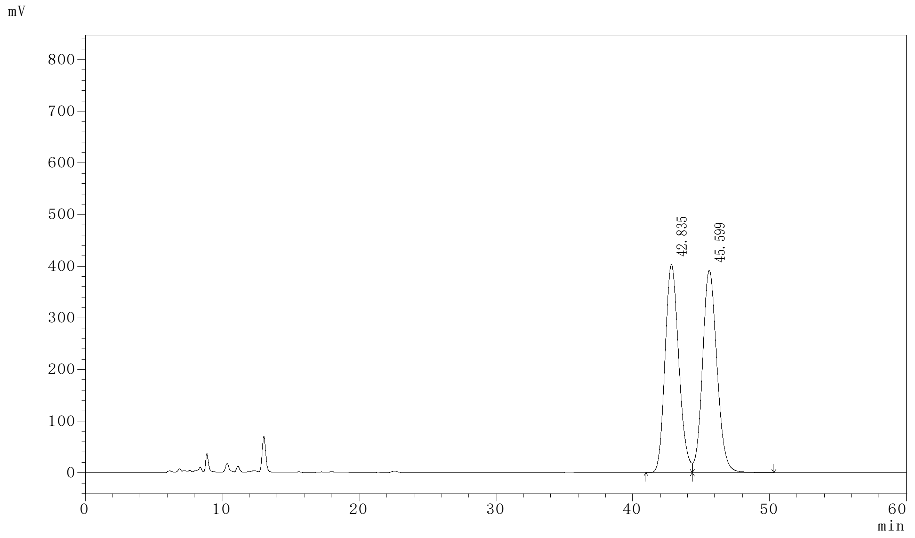

Figure S69. HPLC trace of racemic **4t**

	Retention Time	Area	Height	Area%
1	42.835	28186711	403221	49.487
2	45.599	28771539	392359	50.513
Total		56958250	795580	100.000

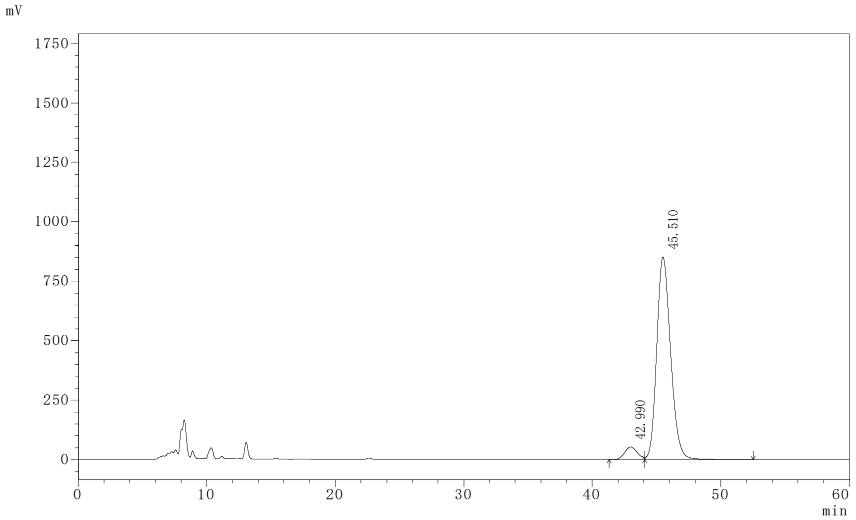

Figure S70. HPLC trace of enantiomeric **4t**

	Retention Time	Area	Height	Area%
1	42.990	3642469	53108	5.388
2	45.510	63956441	851787	94.612
Total		67598910	904895	100.000
